# Aggregating sequences that occur in many proteins constitute weak spots of bacterial proteostasis

**DOI:** 10.1038/s41467-018-03131-0

**Published:** 2018-02-28

**Authors:** Ladan Khodaparast, Laleh Khodaparast, Rodrigo Gallardo, Nikolaos N. Louros, Emiel Michiels, Reshmi Ramakrishnan, Meine Ramakers, Filip Claes, Lydia Young, Mohammad Shahrooei, Hannah Wilkinson, Matyas Desager, Wubishet Mengistu Tadesse, K. Peter R. Nilsson, Per Hammarström, Abram Aertsen, Sebastien Carpentier, Johan Van Eldere, Frederic Rousseau, Joost Schymkowitz

**Affiliations:** 1 Laboratory of Clinical Bacteriology and Mycology, Department of Microbiology and Immunology, KULeuven, Herestraat 49, 3000 Leuven, Belgium; 2 Switch Laboratory, VIB Center for Brain and Disease Research, Herestraat 49, 3000 Leuven, Belgium; 3 Switch Laboratory, Department of Cellular and Molecular Medicine, KULeuven, Herestraat 49, 3000 Leuven, Belgium; 4 Astbury Centre for Structural Molecular Biology, University of Leeds, Leeds, LS2 9JT UK; 5 School of Molecular and Cellular Biology, University of Leeds, Leeds, LS2 9JT UK; 6 Laboratory of Food Microbiology, Department of Microbial and Molecular Systems (M²S), KULeuven, Kasteelpark Arenberg 22, 3001 Leuven, Belgium; 7 Department of Physics, Chemistry and Biology, Linköping University, SE-581 83 Linköping, Sweden; 8 Systems Biology based Mass Spectrometry Laboratory (SyBioMa), KULeuven, Herestraat 49, 3000 Leuven, Belgium

## Abstract

Aggregation is a sequence-specific process, nucleated by short aggregation-prone regions (APRs) that can be exploited to induce aggregation of proteins containing the same APR. Here, we find that most APRs are unique within a proteome, but that a small minority of APRs occur in many proteins. When aggregation is nucleated in bacteria by such frequently occurring APRs, it leads to massive and lethal inclusion body formation containing a large number of proteins. Buildup of bacterial resistance against these peptides is slow. In addition, the approach is effective against drug-resistant clinical isolates of *Escherichia* *coli* and *Acinetobacter* *baumannii*, reducing bacterial load in a murine bladder infection model. Our results indicate that redundant APRs are weak points of bacterial protein homeostasis and that targeting these may be an attractive antibacterial strategy.

## Abstract (web summary)

Aggregation is sequence-specific and nucleated by short aggregating protein segments (APR). Here authors use a multidisciplinary approach to show that in *E.coli* some frequently occurring APRs lead to protein aggregation and ultimately bacterial cell death, which could serve as antibacterial strategy.

## Introduction

Loss of protein homeostasis^1^ is a constant threat for any living cell due to the highly crowded intracellular environment that brings into close proximity a large variety of polypeptides that need to undergo error-prone folding reactions in order to attain their native conformation^2^. To control this threat, cells have evolved a complex network of molecular chaperones, proteases, and other specialized molecules^3^. In spite of these cellular-response mechanisms, human protein-folding pathologies have made it clear that under persistent exposure to aggregating proteins, for example, as a result of mutation, protein homeostasis can eventually break down, which ultimately results in cell death^4^. On the other hand, protein aggregation turns out to be a highly ordered and specific process: aggregation is more efficient between similar than between unrelated polypeptides^5–7^. At a mechanistic level, protein aggregation is mediated by short (between 5 and 15 residues) aggregation-prone sequence segments (called APRs), which on average occur at least once every 100 amino acids in the primary polypeptide sequence^8^. These APRs are generally sequence segments constituting the hydrophobic core of globular proteins or protein–protein interaction interfaces^9^. While forming the most stable part of the native proteins, in unfolded proteins, APRs can also self-assemble with identical APRs from another protein to form β-structured aggregates^10^. The risk of aggregation is thus the highest during translation before the protein attains its native conformation^11^. As the sequence of most APRs is unique within a given proteome^7^, aggregation will generally be restricted to identical proteins. However, a minority of APRs (or close homologs thereof) are found in several and sometimes many different proteins^7^. Given the sequence specificity of aggregation, this suggests that these proteins could coaggregate via such a common APR. The redundancy of these APRs therefore also suggests that they might constitute particularly vulnerable proteomic segments, and that under conditions of stress, these might act as hot spots for the initiation of proteostatic collapse.

In order to test this concept, here, we screen 125 aggregating sequences that have a high degree of redundancy in the *Escherichia coli* proteome. In this manner, one peptide containing this APR could potentially affect the folding of many proteins containing highly similar APRs. Using this strategy, we identify several peptides that efficiently induce bactericidal protein aggregation and inclusion body (IB) formation in *E*. *coli*. This process is bactericidal to *E*. *coli* as well as *Acinetobacter baumannii*, including clinical strains that are resistant to current antibiotics. Analyzing these peptides in *E*. *coli* in more detail using several molecular and proteomic approaches, we find that these peptides induce widespread aggregation of bacterial proteins, resulting in bactericidal aggregation cascades involving hundreds of proteins. Moreover, the peptides effectively reduce bacterial load in a murine bladder *E*. *coli* infection model, suggesting that redundant APRs in bacterial proteomes can be targeted for therapeutic purposes.

## Results

### Redundant aggregating sequences are rare

**Fig. 1:**
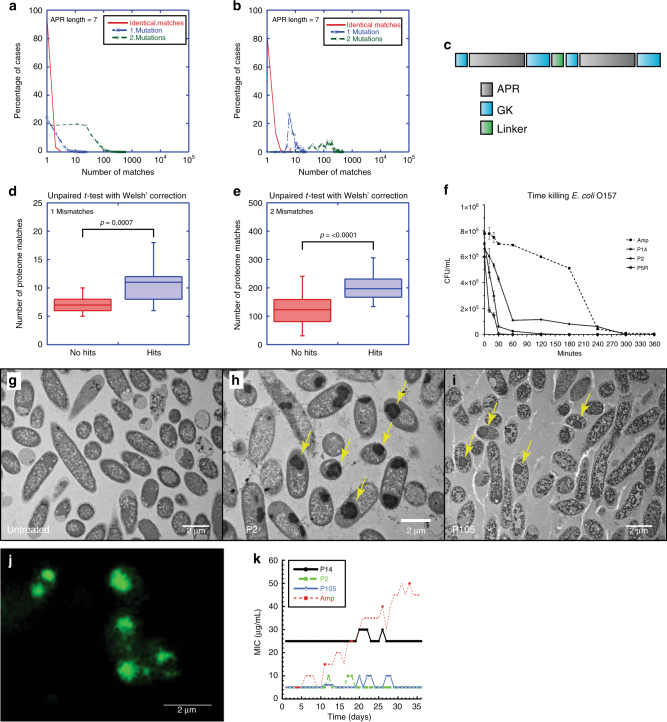
Proteome analysis, design, and screening of redundant APRs. **a** Distribution of the redundancy of APR sequences of length seven in the *E*. *coli* proteome: percentage of identical sequences (red), one mismatch (blue), and two mismatches (green). **b** Same distribution as in **a** for the 75 most redundant APRs in *E*. *coli*. **c** Design pattern for aggregating peptide screen. Tandem APRs are linked by a linker (a single proline residue) and embedded between gatekeeper residues (GK; arginine residues). **d**, **e** APR redundancy for toxic versus nontoxic peptides considering one (**d**) or two (**e**) mismatches. The bottom and top of the boxes are the first and third quartiles, and the band inside the box represents the median. The whiskers encompass the minimum and maximum of the data. Significant differences were computed using Welch’s *t* test. **f** Time-killing curve of selected peptides (P14, P2, and P5R) and ampicillin (Amp) against *E*. *coli* strain O157:H7 treated at MIC concentration (average and SD of three replicates). **g**–**i** Transmission electron microcopy (TEM) of cross-sections of resin-embedded *E*. *coli* O157:H7, treated for 2 h with buffer (**g**), P2 peptide (**h**), and P105 peptide (**i**) at MIC concentration. **j** Wide-field structured illumination microscopy (SIM) image of *E*. *coli* O157:H7 treated with P2 and stained with the amyloid-specific dye pFTAA (0.5 µM). **k** Monitoring of spontaneous buildup of resistance by monitoring the MIC value of *E*. *coli* O157: H7 cultures that are maintained on sublethal doses (50% of MIC) of selected peptides (P14, P2, and P105) or ampicillin (Amp) for 36 days

We used the statistical thermodynamics algorithm TANGO to analyze the aggregation propensity and APR redundancy of the *E*. *coli* strain O157:H7 proteome. This yielded 3,535 APR sequences of at least six amino acids in length with a TANGO score of at least 20%. Given the length limitation of roughly 20 amino acids in solid-phase peptide synthesis with regard to yield and purity, our peptide design imposes a maximum length on the APRs that can be accommodated (APRs will be incorporated in a tandem repeat design peptide comprising twice the APR flanked by three gatekeeper arginine residues and linked by a single proline; see below), forcing us to restrict our experimental analysis to the 1,542 APRs with a length of seven amino acids. To analyze the redundancy of these sequences, we calculated for each of these APRs the number of times their sequence occurred in other proteins in the *E*. *coli* proteome, considering zero, one, or two amino acid mutations (Fig. 1a). This shows that for more than 80% of the seven-residue APRs, their exact sequence is unique in the *E*. *coli* proteome, while virtually no heptameric APR is found in more than five different proteins (Fig. 1a, red line). This observation might be related to the previous finding that selective pressure shapes sequence divergence in repeat-domain proteins such as titin in order to avoid interdomain aggregation^12^. Allowing one mutation in the APR (85% APR sequence identity), we found that the number of unique APRs drops to 20% (Fig. 1a, blue line), but there is almost no heptameric APR sequence that possesses more than 10 APR homologs with a single-point mutation in other *E*. *coli* proteins. This suggests that while one mutation is in many instances probably sufficient to avoid coaggregation (especially hydrophobic to charged-residue mutations), several single-point mutations will still allow for coaggregation (especially conservative hydrophobic mutations), which is confirmed by previous observations^6,13^. Finally, allowing for two mutations (70% APR sequence identity), we found that most APRs have more than 10 homologous APRs (Fig. 1a, green line); this suggests that at 70% sequence divergence (i.e., two mismatches in a heptameric sequence), coaggregation may be a rare occurrence, although it should still not be excluded. Indeed, Fig. 1a (green line) also suggests that high redundancy of two mismatch mutations (above 30) is still to be avoided. Very similar distributions of the number of homologous or identical APRs could also be observed in other bacterial proteomes, including *Klebsiella pneumoniae*, *Pseudomonas aeruginosa*, and *A. baumannii* (Supplementary Fig. 1), suggesting that this is a universal feature of bacterial proteomes.

To experimentally investigate the impact of APR redundancy on the proteostatic robustness of the *E*. *coli* proteome, we ranked APRs at the extreme of the redundancy distribution in Fig. 1a, showing up to 10 matches with one mismatch mutation and up to 100 matches with two mutations, and selected the first 75 most frequently occurring sequences from this list (Supplementary Table 1). The extreme values of these APRs are apparent from Fig. 1b, showing an enrichment of tail values of the *E*. *coli* APR redundancy distribution displayed in Fig. 1a. Counting all amino acid substitutions as equal in terms of their likely β-interaction with the bait sequence is only a very rough approximation, but one that needs to be made since reliably separating amino acid substitutions that are conducive of β-interaction from those that are not is beyond the scope of current prediction algorithms.

### Redundant APRs cause bacterial cell death

In order to generate efficient aggregation seeds, here, we employed a tandem repeat design previously validated^14,15^, in which the redundant APR is incorporated as a tandem repeat separated by a linker constituted by a single proline. In order to increase the colloidal stability of these aggregating peptides, the APRs are flanked by aggregation gatekeepers, a class of residues that was previously shown to reduce aggregation kinetics^8,16,17^. Since positively charged residues have also been shown to help bacterial uptake^18^, we selected arginine to obtain the following peptide layout: R-APR-RR (Fig. 1c). To further modulate the kinetics of aggregation of these peptides, we also added two variants of each of the first 25 peptides in the list by randomly mutating one residue in the first APR repeat to arginine (Supplementary Table 1). These 100 peptides were generated using solid-phase synthesis at 200-nmol scale and dissolved in dimethyl sulfoxide (DMSO) to a theoretical stock concentration of 2 mM (by assuming 100% synthesis efficiency).

As we were looking for peptides capable of inducing a lethal proteostatic collapse, our primary screen consisted of measuring the effect of our peptides on the growth of *E*. *coli* O157:H7 at dilutions of the peptide corresponding to concentrations of 1, 6, 12, and 25 μg/mL. Although no peptide inhibited bacterial growth at the highest dilution, 43 of them inhibited bacterial growth of *E*. *coli* O157 at 25 μg/mL, of which 11 were still active at 12 μg/mL and six had an apparent minimum inhibitory concentration (MIC) value of 6 μg/mL (Supplementary Table 2). We separated the original 75 APRs into two groups based on their inhibitor activity: the hits listed in Supplementary Table 2 and the inactive peptides. We then compared the sequence redundancy in the *E*. *coli* proteome of the APRs in both groups and found that the number of sequence matches found at both one and two mutations distance were significantly higher in the active group than in the inactive group (Fig. 1d, e). This shows that a high APR redundancy is associated to bacterial cell death, suggesting that these sequences could indeed represent proteomic weak spots of susceptibility for proteostatic collapse.

### Bactericidal activity is associated to IB formation

In order to investigate whether APR redundancy does result in proteostatic collapse, we selected resynthesized and high-performance liquid chromatography (HPLC)-purified four positive peptides from the screen, P2, P5, P14, and P105, as well as three negative peptides, P3, P4, and P11, and confirmed their MIC and minimum bactericidal concentration (MBC) values (Table 1). Analysis of the rate of peptide bactericidal activity against *E*. *coli* O157:H7 (at MIC concentration) showed that the peptides exerted full bactericidal effect within 30 min to 2 h (Fig. 1f). Biophysical analysis of the P2 peptide in vitro, using mass spectrometry (MS), dynamic light scattering (DLS), Fourier transform infrared spectroscopy, transmission electron microscopy (TEM), and tinctorial assays confirmed the intrinsic aggregation propensity of the P2 peptide (Supplementary Note 1 and Supplementary Fig. 2). Cross-section TEM of peptide-treated bacteria revealed the widespread presence of large IBs, a hallmark of protein aggregation in *E*. *coli*, suggesting that the peptides act by interfering with bacterial proteostasis (Fig. 1g–i). These IBs, which are also called large polar aggregates^19^, could be stained with pentameric formyl thiophene acetic acid (pFTAA, Fig. 1j and supplementary Fig. 3), an extensively characterized amyloid-specific dye^20–22^, which specifically binds to amyloid-like aggregates as well as disease-associated protein IBs^22^. This IB-staining pattern could be observed for all bactericidal peptides, but not for any of the other peptides, although some other pFTAA-positive structures could be discerned (Supplementary Fig. 3). We confirmed the cross-β-structure content of IB using thioflavin-T staining and correlative atomic force microscopy nanoimaging and Fourier transform infrared spectroscopy (Supplementary Note 2 and Supplementary Fig. 4). These data show that aggregation, resulting in cross-β-structure-enriched IBs, is a crucial property of the bactericidal peptide treatment.

**Table 1:** MIC and MBC values of selected peptides purified by HPLC grade on *E*. *coli* O157

Purified peptide	Sequences	MIC (μg/mL)	MBC (μg/mL)
P2	RGLGLALVRRPRGLGLALVRR	6	6
P2Pro	RGLGPALPRRPRGLGPALPRR	>100	>100
P5	RALLTTLLRRPRALLTTLLRR	6	6
P5R	RRALLTTLLRRPRALLTTLLRR	12	12
P105	RALLRTLLRRPRALLTTLLRR	12	12
P14	RGLLALLARRPRGLLALLARR	6	6

*MIC* minimum inhibitory concentration, *MBC* minimum bactericidal concentration, *HPLC* high-performance liquid chromatography

When bacteria were repeatedly passaged on sublethal concentrations (50% of MIC) of the active peptides for a period of 36 days, no development of resistance was observed, whereas this was the case for the control antibiotic ampicillin (Fig. 1k). These data are supportive of a mode of action involving many targets throughout the *E*. *coli* proteome. To investigate this in more detail, peptide P2 was selected for further analysis. As a control, we generated a variant of P2, called P2Pro, in which we introduced proline substitutions at two positions in the APRs (Table 1), which conserve the hydrophobicity but disrupt the β-sheet propensity and hence reduce the aggregation propensity of the peptides^23^. When we treated bacteria with the control peptides, we obtained MIC values of more than 200 μg/mL, indicating again that β-aggregation is key for the bactericidal effect of P2.

**Fig. 2:**
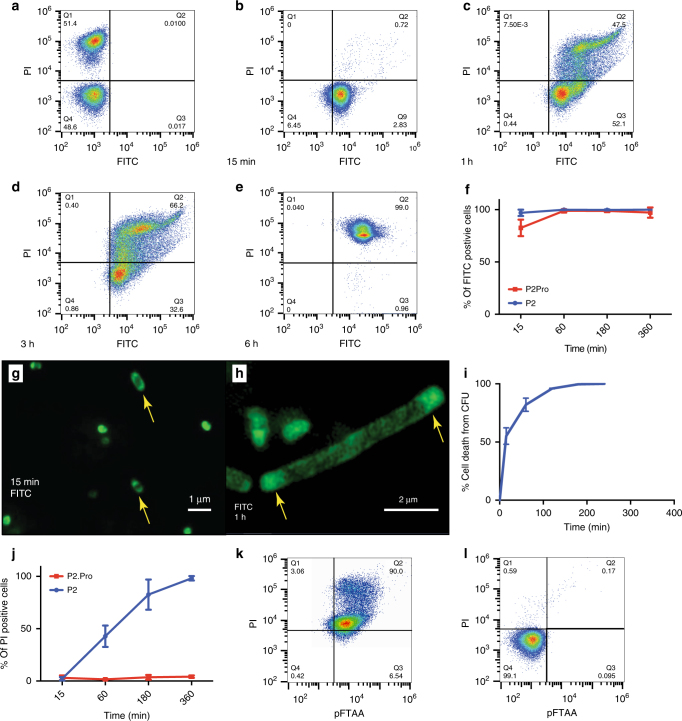
Uptake and inclusion body formation. **a**–**e** Fluorescence-activated cell sorting (FACS) analysis of 40,000 *E*. *coli* O157: H7 cells, measuring FITC fluorescence (*x*-axis) and propidium iodide (PI) fluorescence (*y*-axis) of **a** untreated and heat-inactivated bacteria mixed at a ratio of 1:1 and **b–e** bacteria treated for 15 min (**b**), 1 h (**c**), 3 h (**d**), and 6 h (**e**) with FITC-labeled P2 at MIC concentration. **f** Average population sizes of FITC-positive cells treated with FITC-P2 or FITC-P2Pro from four independent experiments such as those shown in **b**–**e**. **g** Wide-field structured illumination microscopy (SIM) image of *E*. *coli* treated with FITC-P2 for 15 min and **h** for 1 h at MIC concentration. **i** Time-dependent cell death following P2 treatment (1 x MIC) as % CFU/mL, in *E*. *coli* O157:H7. **j** Average population sizes of PI-positive cells (propidium iodide) from four independent FACS experiments such as those shown in **a**–**e**. **k** FACS analysis of 40,000 *E*. *coli* O157:H7 cells, measuring pFTAA fluorescence (*x*-axis) and PI fluorescence (*y*-axis) after 3 h of treatment with P2 at MIC concentration. **l** Same as **h**, but after treatment with 100 μg/mL P2Pro

We derivatized P2 with fluorescein isothiocyanate (FITC) and established that the conjugate retained its antibacterial activity (MIC = 3 μg/mL against *E*. *coli* O157:H7) and quantified P2 uptake over time by flow cytometry (Fig. 2a–f). Analysis of P2 uptake by *E*. *coli* O157:H7 showed that after 15 min, 97.7 ± 2.9% (*N* = 4, mean and s.d.) of the cells are positive for FITC (Fig. 2b, f), increasing to nearly 100% after 1 h and beyond (Fig. 2c–f). In parallel, fluorescence microscopy of treated *E*. *coli* O157:H7 at MIC concentration confirmed no enrichment at the cell membrane of FITC-P2, but rather showed a clear accumulation of fluorescence in intracellular polar IBs from 15 min onward (Fig. 2g) that persisted at later time points (Fig. 2h) and thus confirmed the cross-section TEM images (Fig. 1g–i). Kinetics of bacterial cell death as measured by colony-forming unit (CFU) determination after P2 treatment (Fig. 2i) closely follow peptide internalization and coincide with the appearance of IBs after 15 min of treatment (50% after 15 min). On the other hand, bacterial cell death as monitored by propidium iodide (PI) uptake as a result of membrane permeabilization increased more slowly (2.1 ± 1.3% after 15 min to 85 ± 13.2% after 3 h, Fig. 2a–e, summarized in Fig. 2j, *N* = 4). This was confirmed by morphological analysis using scanning electron microscopy (SEM) (Supplementary Note 3 and Supplementary Fig. 5).

Together, these data demonstrate that P2 uptake and IB formation occur in close succession, and this is followed by cell death. Cell death precedes membrane disruption as significant growth inhibition is established coincidentally with IB formation but before membrane permeability or deformation can be observed. Importantly, all PI-positive cells stain positive for aggregation by pFTAA (Fig. 2k) and when bacteria were treated with FITC-P2Pro, which showed comparable uptake to P2 (Fig. 2f), no protein aggregation ensued (Fig. 2l) and hence no cell death could be detected (Fig. 2l). This again shows that aggregation of the peptide is essential to mediate its bactericidal effect.

### Protein aggregation is required for cell death

**Fig. 3:**
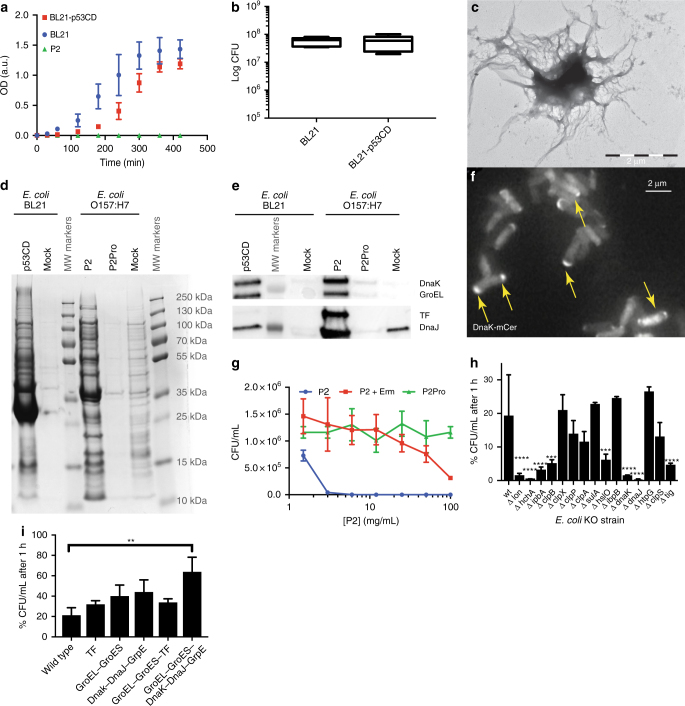
Inclusion body formation and proteostatic collapse. **a** Growth curve of *E*. *coli* BL21-overexpressing p53CD (red) and control in the presence (green) or absence (blue) of P2 (average and SD of three replicates). p53CD bacterial growth in the presence of 0.4 mM IPTG. **b** Colony formation by *E*. *coli* BL21 p53CD-overexpressing bacteria. The bottom and top of the box are the first and third quartiles, and the band inside the box represents the median. The whiskers are drawn using Tukey’s method and show the extreme values that fall within 1.5 times the interquartile range. **c** Transmission electron microscopy image of an inclusion body from P2-treated *E*. *coli* O157:H7 (uranyl acetate). **d** Representative Coomassie blue SDS-PAGE of inclusion bodies from *E*. *coli* BL21-overexpressing p53CD (lane 1), mock (lane 2), and *E*. *coli* O157:H7 treated with P2 (lane 4), P2Pro (lane 5), or DMSO (lane 6). Molecular-weight markers are shown in lanes 3 and 7. **e** Western blot for dnaK, groEL, tig, and dnaJ of the same samples than that in **d**. **f** Fluorescence microscopy image of *E*. *coli* cells stably expressing a fluorescent fusion of DnaK (mCer) treated with P2 at MIC concentration. **g** Growth inhibition of cells treated with P2 with/without erythromycin (Erm, 100 μg/mL, average and SD of three replicates). **h** Percent of colony-forming units after treating bacterial KO strains (KEIO) for 1 h with P2 at its MIC concentration. **i** Percent of colony-forming units of chaperone-overexpressing *E*. *coli* strains treated by P2 peptide at MIC concentration for 1 h. Significant differences from the WT are calculated using ordinary one-way ANOVA and Dunnett’s multiple-comparison test. Statistical significance is indicated as follows: **P ≤ 0.01, ***P ≤ 0.001, ****P ≤ 0.0001

Bacterial IB formation is a common event associated with cellular stress including exposure to heat and, perhaps most famously, recombinant protein (over)expression^24^. This process, however, is often transient and reversible and does not necessarily lead to bacterial cell death. In fact, recombinant protein production in bacteria relies to a large extent on the ability of bacteria to cope with IBs. As an example, we measured the consequences of overexpressing the highly aggregation-prone core domain of the human p53 protein (p53CD) on growth (Fig. 3a) and colony formation (Fig. 3b) of *E*. *coli* BL21 cells, which are routinely used for recombinant protein production. Although p53CD expression resulted in a delay of the exponential growth phase, consistent with cellular stress resulting from overexpression, there was no effect on colony formation, showing that the stress in this condition is not lethal. In order to understand why P2-induced IB formation is irreversibly toxic, we compared the composition of IBs purified from *E*. *coli* O157:H7 cells treated with P2 at MIC concentration for 1 h with IBs purified from *E*. *coli* strain BL21 overnight overexpressing p53CD. Inspection of the resulting samples by TEM confirmed the successful purification of these IBs (Fig. 3c). The composition of IBs was subsequently analyzed by Coomassie-stained sodium dodecyl sulfate-polyacrylamide gel electrophoresis (SDS-PAGE) (Fig. 3d). The overall pattern of Coomassie staining revealed that a large number of similar bacterial proteins are trapped in the IBs of both P2-treated *E*. *coli* O157:H7 and p53CD-overexpressing *E*. *coli* BL21, but not in untreated bacteria, suggesting a common molecular machinery associated with IB formation. In the p53CD IBs, the band corresponding to the molecular weight of p53CD and western blot is clearly visible using the p53 mouse monoclonal antibody pAb240 that recognizes a linear epitope located in the p53CD construct^25^ (Supplementary Fig. 6). Among the proteins trapped in both types of IBs, a number of molecular chaperones that are known to occur in IBs^26^ could be detected, including the bacterial heat shock protein 70 (Hsp70) homolog DnaK, the Hsp60 chaperonin GroEL, the ribosome-associated chaperone trigger factor (TF), and the bacterial Hsp40 DnaJ (Fig. 3e). The polar localization of a fluorescently traceable DnaK-mCerulean3 fusion protein (the latter moiety comprising a blue fluorescent protein) in *E*. *coli* K-12 MG1655 cells exposed to P2 confirms the association of DnaK with IBs (Fig. 3f).

We then investigated the Coomassie-stained SDS-PAGEs of IBs isolated both from *E*. *coli* O157:H7 and BL21:DE3 upon treatment with the remaining active and inactive peptides, which showed a similar high-intensity pattern of IB-associated proteins for the bactericidal peptides, which was absent or much reduced in the inactive peptides (Supplementary Fig. 7). So, when peptides with redundant APRs successfully induce aggregation in the cell, the process is bactericidal, but when a similar degree of aggregation is caused by the overexpression of a protein that is alien to this strain, it is not significantly toxic. These observations show that any toxicity that is associated with IB formation depends on the proteins that are aggregating into the IBs: a heterologous protein that aggregates does not represent a loss of an essential cellular function, and is hence not likely to be toxic. On the other hand, the simultaneous aggregation of many of the bacterial cell’s own proteins would eventually be expected to accumulate such high and pleiotropic levels of loss of function that cellular viability is ultimately irreversibly impaired. As a confirmation, we generated a tandem peptide that follows our design pattern, but that instead of a bacterial fragment contains an APR from p53CD and indeed found this not to be toxic to *E*. *coli* (Supplementary Note 4, Supplementary Figs. 7 and 8). This shows that aggregating peptides of this design are not toxic per se, but that their bactericidal effect depends on the induction of the aggregation of cellular proteins.

### Cotranslational loss of protein homeostasis

To gain more insight into the specific composition of IBs associated with the bactericidal activity of P2, we performed MS proteomic analysis. To achieve the highest possible coverage, we combined shotgun analysis of the entire IB samples with samples obtained from sectioning SDS-PAGE gels of P2-induced IBs into five equal sections and analyzed the protein composition of each by MS. We analyzed six independent biological repeats and considered them as hit proteins that were detected with a confidence of 99% in at least two of the six samples (Supplementary Data 1).

This analysis showed that, in agreement with the western blot, a wide range of chaperones can also be detected in the MS data. This included the cotranslational TF, the chaperonin system groEL/groES, the Hsp70 system dnaK/dnaJ/grpE, the hsp90 homolog htpG, and the small Hsp ibpA, as well as the proteases lon and clpX/clpP/clpA (Supplementary Data 1). In total, 541 proteins were detected in the P2-induced IBs, suggesting that the bactericidal impact of P2 treatment corresponds to an extensive proteome-wide aggregation of proteins, in line with our initial design that aimed at inducing the collapse of protein homeostasis by aggregation of multiple proteins. Apart from the chaperones, the IBs were strongly enriched in ribosomal proteins (NCBI DAVID^27^, 47 genes, enrichment score of 26.12, Benjamini *P* value < 10^−98^), indicating that protein aggregation in response to P2 may occur cotranslationally. To verify this hypothesis, we measured the MBC value of P2 in the presence of the macrolide antibiotic erythromycin, which is a bacteriostatic drug that acts by blocking the polypeptide exit channel in the ribosome. We observed a marked desensitization of bacteria (*E*. *coli* O157) (MBC > 100 μg/mL) to P2 after pretreating the cells with 100 μg/mL erythromycin for 2 h to block translation during peptide exposure, strongly supporting cotranslational induction of protein aggregation by P2 (Fig. 3g). In line with this, we observed by fluorescence-activated cell sorting (FACS) that there was no buildup of pFTAA staining in the P2-treated bacteria in the presence of erythromycin (Supplementary Fig. 9), even though the uptake of FITC-P2 was not impaired by the presence of the ribosome inhibitor (Supplementary Fig. 10). These data show that in the absence of protein translation, there is no induction of protein aggregation and that this eliminates the bactericidal effect, again showing that the peptide needs to induce protein aggregation in the bacteria to mediate cell death. The data in Supplementary Fig. 9 additionally demonstrate that the contribution of P2 itself to the pFTAA staining is small compared to the bacterial proteins that aggregate in the IBs, meaning that the lack of pFTAA staining in cells treated with P2Pro (Fig. 2l) really comes from the failure of this peptide to induce aggregation of bacterial proteins. In combination, the effect of erythromycin on P2 and the comparison of P2 with P2Pro establish a causal link between cotranslational induction of the aggregation of many bacterial proteins and the bactericidal effect of P2. Moreover, we could detect eight proteins that contain a sequence fragment similar to the APR of P2, including the HcaB protein from which the sequence was derived, and could integrate the other proteins in the IBs as nodes in an aggregation network connected by sequence-specific coaggregation edges (Supplementary Note 5, Supplementary Table 3, Supplementary Figs. 11, 12, 13, 14, and Supplementary Data 1).

If the bactericidal effect of P2 is indeed mediated by a loss of protein homeostasis, the cellular chaperone machinery would be expected to counteract or limit the effect of the peptide. In order to evaluate this, we determined the effect of P2 on 18 gene deletion strains for major bacterial chaperones and proteases in the *E*. *coli* K-12 BW25113 strain (taken from the KEIO collection^28^) and found that from the individual knockouts of the principal proteostatic components of *E*. *coli*, only the hsp70 *dnaK* and the small Hsp, hsp31, had a mild effect on the MIC value of P2 (Table 2). To ensure that this effect was not due to the reduced overall viability of the *dnaK* deletion strain, we also tested the MIC value at 30 °C and obtained the same result (MIC = 6 μg/mL). This confirms that the direct inhibition of chaperones and proteases is not the principal mechanism of action of P2. However, in several of the chaperone or protease deletion strains, the percent of bacterial cell survival after 1 h was significantly decreased for several deletion strains compared to the wild-type strain (Fig. 3h), showing that cell killing occurred much faster in the absence of certain chaperones. The deletion strains with the strongest effect largely matched those found in the IBs by MS, including those of the cotranslationally acting chaperones TF (*tig*), *dnaK* (Hsp70 family), and its cochaperone *dnaJ*, as well as the small Hsps *hchA* (Hsp31), *hslO* (Hsp33), and *ipbA* (α-crystallin family). The disaggregase *clpB* and the aggregation-controlling protease *lon* also had strong effects. A similar picture emerged when we analyzed the sensitivity of *E*. *coli* BL21 after overexpression of selected chaperones: there was no effect on the MIC values, but the cell killing was slowed down compared to the wild-type strain in this experiment when the groEL/ES and dnaK/J/grpE systems were expressed in combination (Fig. 3i & Table 3). This demonstrates that the protein quality control (PQC) machinery temporarily opposes the aggregation induced by the P2 peptide, but is eventually overwhelmed and ends up associated to the aggregated proteins in the IB fraction.

**Table 2:** MIC of P2 for chaperone deletion strains

Gene deletion	MIC (μg/mL)	Protein name	Description
KEIO WT	12		
∆ *clpP*	12	ClpP	Proteolytic subunit of the Clp protease
∆ *clpA*	12	ClpA	Substrate-specifying adapter for the Clp protease
∆ *clpS*	12	ClpS	Specificity adapter for the Clp protease (binds to and modulates ClpA)
∆ *clpX*	12	ClpX	ATP-binding subunit of the Clp protease
∆ *lon*	12	Lon	ATP-dependent protease, required for suppression of aggregation
∆ *sulA*	12	SulA	Suppressor of Lon
∆ *clpB*	12	ClpB	Disaggregase of the Hsp100 family
∆ *dnaK*	6	DnaK	Folding chaperone of the Hsp70 family
∆ *dnaJ*	12	DnaJ	Cochaperone to DnaK of the Hsp40 family
∆ *grpE*	n/a	GrpE	Nucleotide exchange factor for DnaK
∆ *htpG*	12		Folding chaperone of the Hsp90 family
∆ *groL*	12	GroEL	Folding chaperone of the Hsp60 family
∆ *groS*	n/a	GroES	Cochaperone of Hsp60, of the Hsp10 family
∆ *hslO*	12	Hsp33	Oxidative stress-induced holdase
∆ *hchA*	6	Hsp31	Heat-dependent and temperature-stress-dependent holdase
∆ *ibpA*	12	IbpA	Small Hsp of the α-crystallin family
∆ *ibpB*	12	ibpB	Small Hsp of the α-crystallin family
∆ *tig*	12	Trigger factor	Cotranslational folding chaperone, ribosome-associated

*MIC* minimum inhibitory concentration, *WT*wild type, *Hsp* heat shock protein

**Table 3:** MIC of P2 after chaperone overexpression in BL21

Plasmid	Overexpressed chaperone(s)	MIC (μg/mL)
pG-KJE8	dnaK-dnaJ-grpE groES-groEL	25
pGro7	groES-groEL	25
pKJE7	dnaK-dnaJ-grpE	25
pG-Tf2	groES-groEL-tig	25
pTf16	tig	25
wt	—	25

*MIC* minimum inhibitory concentration

### IB composition versus toxicity

To better understand the importance of the quantity and identity of proteins pulled into aggregates in distinguishing between lethal aggregation and controlled, nontoxic IB formation, we performed a large-scale MS proteomics experiment, comparing IB composition from control conditions to those formed upon overexpression of p53CD with treatment with toxic (P2, P5, and P14) and nontoxic peptides (P2Pro, P4), as detailed in Supplementary Note 6, Supplementary Data 2 and 3, and Supplementary Fig. 15. The first observation that stands out in this analysis is that IBs associated with lethal conditions contain significantly more proteins than those associated to nontoxic conditions (Supplementary Fig. 16A). A common core of IBs was defined by identifying a large group of 424 proteins that are present in IBs of untreated, as well as toxic and nontoxic peptide-treated BL21. This common core appears to be primarily composed of the molecular machinery required to mediate and control IB formation, as well as other proteins that appear to associate to IBs for reasons that are less obvious (Supplementary Fig. 17A and B). The former category comprises the molecular chaperones, such as the chaperonin *groE*, the bacterial hsp70 *dnaK*, the disaggregase *clpB*, the cotranslational foldase TF, and, to a lesser extent, the bacterial Hsp90 *htpG* among others (Supplementary Fig. 18).

All structural constituents of the ribosome and other elements involved in the control of protein translation also commonly dominate the formation of IBs irrespective of the peptide treatment (Supplementary Fig. 17C and D). However, in addition to this common core of proteins, each IB contains an additional set of polypeptides specifically associated to each condition (Supplementary Fig. 14B). Of all the samples, the IBs resulting from the overexpression of the p53CD protein contain the smallest number of additional proteins (eight proteins, including p53CD). The p53CD polypeptide strongly dominates the composition of the IBs from the overexpressing cells (Fig. 3d). This indicates that the heterologous expression of p53CD leads to IBs that essentially consist of a large quantity of the overexpressed protein, plus the proteins typically found in the IB fraction across conditions. This makes sense in the context of recombinant protein purification from such IBs and explains the lack of toxicity observed under these conditions. It is also in line with the notion that p53CD aggregation constitutes a proteostatic stress that, contrary to P2, does not cause a proteostatic collapse and is not lethal to the bacterial cell. In the IBs from nonlethal conditions, including p53CD, P2Pro, and P4, we detect fewer additional proteins than in the IBs from lethal conditions (Supplementary Fig. 16B). Moreover, these additional proteins in the nonlethal IBs are often from the same functional categories as the GO enrichments associated to the common core, suggesting that the formation of nontoxic IBs is a more controlled process than those formed in the lethal conditions (Supplementary Fig. 17). In the IBs from lethal conditions combined (P2, P5, and P14), we found between 47 and 154 bacterial proteins in addition to the typical IB proteins found in the common core, with contrasting molecular functions (Supplementary Fig. 17C), showing what a devastating impact these peptides have on the PQC, and confirming the notion that peptides containing redundant APRs cause widespread protein aggregation. The isolated genetic deletion of many of these elements individually completely impairs viability. So, it may be unsurprising that the accumulated loss of many of these proteins is lethal, even if the knockdown of each individual protein is likely to be less complete than in the genetic deletion.

In conclusion, the MS data presented here are in good agreement with the notion that our peptides cause a pleiotropic and accumulated loss of function to protein aggregation that is eventually lethal.

### Exploiting redundant APRs in vivo

Since bioinformatics analysis showed that our peptides differ markedly from existing antimicrobial peptides (Supplementary Note 7), we wanted to know if the induction of proteostatic collapse mediated by P2 could be exploited as an antimicrobial peptide.

**Fig. 4:**
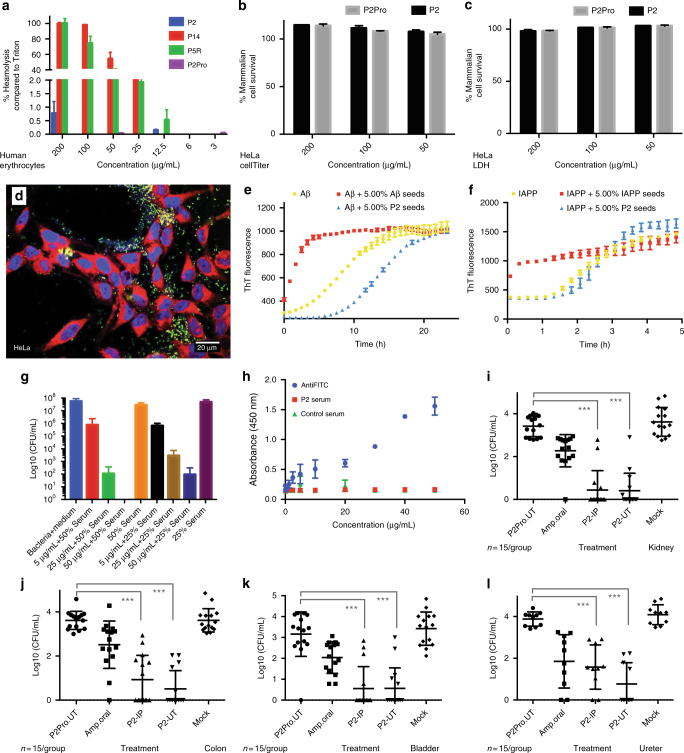
Cross-seeding and in vivo activity. **a** Concentration-dependent hemolysis of human erythrocytes by selected peptides (average and SD of three replicates) shown as percent of hemolysis compared to 1% Triton. **b**, **c** Cytoxicity of P2 (black bars) and P2Pro (gray) to human HeLa cells measured using the CellTiter Blue assay (**b**) (average and SD of three replicates) and the lactate dehydrogenase (LDH) release assay (average and SD of three replicates), represented as percentage of cell survival compared to control. **d** Fluorescence micrography of HeLa cells mixed with *E*. *coli* O157:H7, treated with FITC-P2 (green channel). Blue is DAPI (4',6-diamidino-2-phenylindole), red is CellMask Deep Red. **e** Aggregation kinetics of the Alzheimer β (Aβ) peptide at 50 µM with/without P2, monitored using thioflavin-T fluorescence (average and s.d. of three replicates). **f** Same as **b** for human islet amyloid polypeptide (IAPP). **g** Inhibitory effect of 5, 25, and 50 µg/mL P2 on bacterial growth in the presence of human blood serum (25 or 50%; average and SD of three replicates). **h** ELISA on immobilized FITC-P2 using blood serum of mice treated for 18 days with 30 mg/kg P2. An anti-FITC antibody was used as a positive control for peptide immobilization (three replicates from three mice). **i–l** Antibacterial efficacy of P2 in a mouse model of bladder infection. The bacterial load of mice infected with *E*. *coli* O157:H7 transurethrally was determined after treatment with P2 (P2 administered urethrally (P2 UT) or intraperitoneally (P2 IP)) and controls (ampicillin administered orally (Amp.oral), buffer (mock), and P2Pro administered urethrally (P2Pro2.UT)) in **i** kidney, **j** colon, **k** bladder, and **l** ureter. Each treatment group consisted of 15 animals. Bacterial loads are expressed as log_10_(CFU/mL). See text and Methods for more details. Plots **i**–**l** show individual measurements, as well as mean and s.d. Significant differences were calculated using ANOVA with Tukey's post hoc test. Statistical significance is indicated as follows: ***P ≤ 0.001

We tested the uptake of P2 in other bacterial strains and found peptide uptake in several different bacterial species (Supplementary Fig. 19). Then, we proceeded to test the activity of P2 against 17 clinical isolates of *E*. *coli* and 15 of *A*. *baumannii*, which displayed a varying spectrum of resistance against well-established antibiotics (Supplementary Table 4). We found P2 to be effective on 16 out of 17 of the tested *E*. *coli* strains and 14 out of 15 of the *Acinetobacter* strains, including those resistant to the last-resort carbapenems. As a first indication of specificity of the peptides, we demonstrated that there were no hemolytic-to-human erythrocytes (Fig. 4a), suggesting that the bactericidal effect of P2 is not the result of a generic toxicity. This was further confirmed by CellTiter Blue (Fig. 4b) and lactate dehydrogenase (LDH) release (Fig. 4c) assays to assess the cytotoxicity of the peptide for HeLa cells. The specificity of P2 for *E*. *coli* O157:H7 was estimated by determining the concentration at which bacterial growth is 50% inhibited (IC50 = 1.5 μg/mL) and compared to the concentration at which the peptide induces 50% lysis of human erythrocytes (LC50 = 1,100 μg/mL), yielding an apparent therapeutic ratio of 730. In order to test the in vivo potential of P2, we treated a coculture of mammalian (HeLa) cells and *E*. *coli* O157:H7 with P2 and observed the preferential accumulation of P2 in bacteria but not in mammalian cells (Fig. 4d). In addition, we tested the cross-reactivity of P2 aggregation with known disease-associated amyloidogenic peptides in vitro by spiking P2 into freshly dissolved preparations of the human Alzheimer β (Aβ) peptide or the human islet amyloid polypeptide and did not observe an increase of the rate of aggregation of these peptides (Fig. 4e, f), showing that the peptide is not a general inducer of protein aggregation. We did, however, observe a delay in the aggregation onset of the Aβ peptide, suggesting that a transient interaction did take place (Fig. 4e). Finally, we found that P2 incubated in 25 or 50% human serum for 2 h was still able to inhibit bacterial growth at 25 and 50 μg/mL (Fig. 4g). Moreover, initial experiments outlined in Supplementary Note 8 showed that mice tolerated P2 treatments well (Supplementary Tables 5, 6 and Supplementary Figs. 20, 21, and 22 and Fig. 4h).

Based on these observations, we tested the antibacterial efficacy of the P2 in a mouse bladder infection model. In this model, an inoculum of 50 μL of a 10^8^ CFU/mL suspension of *E*. *coli* O157:H7 was delivered via the urethra to the bladder of healthy Swiss mice. One hour post infection, we administered a single injection of P2 at 10 mg/kg, either via the urethra (denoted at UT, *n* = 15) or intraperitoneally (denoted as IP, *n* = 15). As a positive control, we included ampicillin treatment, which was administered orally (20 mg/kg), while P2Pro was administered urethrally (10 mg/kg) and buffer treatment served as negative controls. Twenty-four hours after treatment, the animals were killed and the bacterial titer in kidney, colon, bladder, and ureter was determined by plating the macerated tissue (Fig. 4i–l). These experiments revealed a significant reduction of the bacterial titer in the different organs of P2-treated animals (*P* value <10^−4^ compared to buffer control and *P* value <10^−4^ compared to nonaggregating P2Pro control, analysis of variance (ANOVA) with Tukey's posttest). The log-fold reduction of the average bacterial load ranged from 2.3 in the ureter to 3.0 in the kidney after IP delivery and from 2.6 in the colon to 3.1 in the ureter upon UT delivery. The effect was comparable to orally dosed ampicillin (20 mg/kg) in the ureter, but P2 treatment was better in reduction of the bacterial load in the other tissues, ranging from 1.48 log-fold in the colon to 2.0 log-fold in the bladder. These results clearly indicate that the antimicrobial activity of P2 against *E*. *coli* is maintained in vivo.

## Discussion

The emergence of multidrug-resistant Gram-negative infections represents one of the major healthcare challenges of the coming decade(s), but alternative treatment options are currently not available^29^. We present here a class of peptides with a strong bactericidal effect against multidrug-resistant clinical isolates of *E. coli* and *A. baumannii*, both in vitro and in a bladder infection model in the mouse. These peptides act by inducing widespread protein aggregation in these bacteria, eventually causing cell death by overcoming the bacterial protein homeostasis system. Protein misfolding and aggregation are relatively common events under normal physiological conditions and are increased under conditions of stress such as heat shock, but the PQC controls the process and avoids that it degenerates. For example, upon recombinant production of heterologous proteins in bacteria, the aggregating protein is stored into IBs, and little or no toxicity is associated. However, human protein aggregation diseases revealed that when stress is too intense or sustained, the capacity of the PQC to control misfolding events is exceeded, resulting in protein aggregation and IB formation^30–32^. Here, we exploit this concept to induce toxic protein aggregation in the Gram-negative bacteria by using aggregation-prone peptides whose sequence is based on aggregation protein sequences that occur in many bacterial proteins. The idea is that these peptides will cause aggregation of many different bacterial proteins that share this short seven-amino-acid stretch. The pleiotropic loss of function of many proteins at the same time eventually overcomes the capacity of the PQC to correct the problem and the viability of the cell is negatively affected. Because the approach disrupts an essential process by targeting many different proteins, we hope that the emergence of resistance may be inherently more difficult for the bacteria than for single-target approaches. Indeed, repeated passaging of bacteria on sublethal concentrations of the active peptides for a period of 36 days did not result in the development of resistance contrary to the control of the antibiotic ampicillin.

Our method is based on the notion that protein aggregation is a sequence-specific process that is nucleated by, and can thus be induced with, short APRs within a protein that self-assemble to form aggregates^8,33–35^. Most proteins possess at least one APR in their sequence. We recently demonstrated, however, that most of these aggregation-prone sequences are unique in a proteome^7,10^. In other words, when a protein aggregates, it will generally only aggregate with identical proteins. We previously exploited the fact that aggregation is sequence specific and that most aggregating sequences are sparse in a proteome to induce specific protein knockdown of target proteins in plants^15^ and mammalian cells^23^, or to achieve protein detection in western blot using protein-specific APRs^7^. During these exercises, we realized, however, that a minority of aggregation-prone sequences are found within several and sometimes many proteins. By the same mechanistic reasoning, this suggests that a minority of proteins will, when aggregating, induce the aggregation of several and even many other proteins. This also suggests that most proteomes possess proteostatic weaknesses that might constitute hot spots for proteostatic collapse under conditions of stress.

From a small screen of 75 frequently occurring APRs from *E*. *coli*, we found that more than half had antibacterial activity at 25 μg/mL, showing that these APRs are a particularly rich source of APRs that can induce widespread protein aggregation. For several of these peptides, we demonstrated that they indeed enter cells and cause protein aggregation in the form of IBs that contain hundreds of bacterial proteins. Taken together, our findings suggest that redundant APRs (which are a minority of the APRs in the *E*. *coli* proteome) indeed represent hot spots for proteostatic collapse, the aggregation of which is so widespread that it is bactericidal. This approach could therefore also represent an interesting paradigm to be explored for the development of a new class of antibiotics.

## Methods

All primer sequences are listed in Supplementary Table 7.

### Bioinformatics analysis

Protein sequences for various bacterial strains were obtained from UniProt^36^, and redundance was removed using the cd-hit algorithm^37^. We employed the software algorithm TANGO to idenitify APRs across this work, using a TANGO score of 5 per residue as the lower threshold. This was previously shown to yield a Mathews correlation coefficient of 0.92^38^ between experimentally determined and predicted aggregation. The parameter configuration TANGO was temperature at 298 K, pH at 7.5, and ionic strength at 0.10 M.

### Peptide synthesis

Initial peptide screens were obtained as microscale peptide sets (200-nmol scale) from JPT (Berlin, Germany). Peptide hits were reordered from several vendors (Genscript, Shanghai, China and PepScan, Lelystad, The Netherlands) at higher purity (>90%) and were also produced in-house using the Intavis Multipep RSi automated synthesizer. In-house HPLC purification was performed with a Zorbax SB-C3 semi-preparative column (Agilent, USA) installed on a Prominence HPLC (Shimadzu, Japan). Peptides were freeze-dried and stored at −20 °C prior to use.

### Bacterial strains and growth conditions

Bacterial cells were collected from different human clinical samples, and from the University Hospital Leuven-Gasthuisberg.

Gram-negative bacterial strains were cultivated in Luria-Bertani (LB) broth (Difco) and Gram-positive bacteria strains were grown in a rich medium, brain heart infusion broth (Difco, Sparks, MD, USA) at 37 °C. Whenever required growth media  were supplemented with appropriate antibiotic to the medium or plates (ampicillin 25 µg/mL, erythromycin 100 µg/mL, chloramphenicol 20 µg/mL, kanamycin 30 µg/mL, l-arabinose 0.5 mg/mL, and tetracycline 2ng/mL). *Escherichia coli* DH5α (Thermo Fisher Scientific) was used for cloning and plasmid amplification. For selection of antibiotic resistance colonies, *E*. *coli* carrying plasmids was grown in LB medium supplemented with the relevant antibiotic. Bacterial CFU counting was done on blood agar plates (BD  Biosciences) or LB agar plates. Species identification and antibiograms for all clinical isolates were performed using MALDI-Tof and VITEK^®^ 2 automated system (bioMérieux).

### MIC determination

The MICs of active peptides were determined via the Broth microdilution assay according to the EUCAST guideline, which was performed in 96-well polystyrene flat bottom microtiter plates (BD Biosciences). Briefly, a single colony was inoculated into 5 mL LB medium and grown to the end-exponential growth phase in a shaking incubator at 37 °C. Cultures were subsequently diluted to an OD_600_ (optical density) of 0.002 (1 × 10^8^ CFU/mL) in fresh LB medium. One hundred microliters of LB medium with different concentration of peptides ranging from 100 to 0.75 µg/mL were serially diluted to the sterile 96-well plate (at least three wells in each plate). Afterwards, 100 µL of the diluted bacteria were pipetted into 96-well plates containing different concentration of peptides. In each plate, the grown bacteria with the maximum concentration of carrier and medium were considered as positive and negative controls, respectively. Thereafter, 96-well plates were statically incubated overnight at 37 °C to allow bacterial growth. OD was measured at 590  nm for 1 s using a multipurpose ultraviolet–visible plate reader, and the absorbance of the growth bacteria was measured using a Perkin Elmer spectrophotometer (1420 Multilabel Counter Victor 3).

### Antibody and antibiotic product codes

The antibodies and antibiotic product codes used are as follows: anti-CLPB (Aviva, catalog# ARP53790_P050) 0.5 µg/mL, anti-DnaK (Aviva, catalog# OAED00201) 1 µg/mL, anti-TF (Clontech, catalog# M201) 2 µg/mL, anti-groEL (Abcam, catalog# ab82592) 1 µg/mL, and anti-DnaJ (Enzo Life Sciences, catalog# ADI-SPA-410-D) 0.5 µg/mL. Ampicillin sodium, CAS number 69-52-3 (Duchefa Biochemie, catalog# A0104), erythromycin, CAS number 114-07-8 (Sigma-Aldrich, catalog# E5389), chloramphenicol, CAS number 56-75-7 (Duchefa Biochemie), and kanamycin CAS number 56-75-7 (Duchefa Biochemie).

### Biophysical characterization

DLS measurements were performed at a ambient temperature using a DynaPro DLS plate reader (Wyatt, Santa Barbara, CA, USA), employing a 830 nm laser at 90° angle in flat-bottomed 96-well microclear plates (Greiner, Frickenhausen, Germany). Data were recorded in 10 s reads and 40 readings were averaged. All calculations of hydrodynamic radius were performed using the Wyatt Dynamics software. For attenuated total reflection Fourier transform infrared spectroscopy (ATR-FTIR), we used the Bruker Tensor 27 infrared spectrophotometer and its Bio-ATR II accessory. We used a spectral resolution of 4 cm^−1^ and recorded spectra in the 900–3,500 cm^−1^ interval, averaging over 120 data acquisitions while purging the instrument with dry air. Atmospheric interference corrections and baseline subtractions were carried out before the spectra were rescaled in the amide II area (1,500–1,600 cm^−1^). For TEM, samples were adsorbed to carbon-coated Formvar 400-mesh copper grids (Agar Scientific) for 1 min, washed, and stained with 1% (wt/vol) uranyl acetate. Electron micrographs were recorded using a JEOL JEM-1400 microscope (JEOL, Tokyo, Japan) at 80 kV.

### Time-killing kinetic assay

The time-killing kinetic study of the peptides was carried out to assess the killing rate of the bacteria at enough exposure time points. This study was done according to standard guide for assessment of antimicrobial activity using time-killing kinetic procedure. Selection of agent concentrations was guided by MIC endpoints.

Briefly, 20 µL of frozen cultures of *E*. *coli* O157: H7 were inoculated into 5 mL LB and grown to the end-exponential growth phase in a shaking incubator at 37 °C. Cultures were subsequently diluted to an OD_600_ = 0.002 (1 × 10^8^ CFU/mL) in fresh LB medium. To evaluate the effect of aggregators over time, bacterial cells were subjected to a concentration of different peptides at the MIC value for different periods of time (5 min, 10 min, 30 min, 1 h, till 6 h). After the defined contact period, 50 µL of each culture was serially diluted and plated on blood agar plates. Plates were incubated overnight at 37 °C without shaking. The number of viable organisms was counted as CFU/mL.

### Multistep resistance development study

The ability of the target strains to develop resistance to active compounds was evaluated by repeated subculturing in the presence of the half-MIC value of the active peptides over 30 days. Briefly, *E*. *coli* O157 cultures were grown in LB medium, the OD of bacteria was then adjusted to an OD_600_ of 0.002 (equivalent to 1 × 10^8^ CFU/mL). Bacterial cells were treated by the aggregator at half-MIC concentration; after a 24 h incubation period, the MIC’s were tested by a microdilution assay according to the EUCAST guideline and the bacteria were re-cultured in the presence of the half-MIC value of the respective aggregator. Ampicillin was used as the positive control in this experiment.

### Scanning electron microscopy

For SEM, *E*. *coli* O157 or BL21 bacterial cells in end-exponential growth phase were diluted to a density of 10^8^ CFU/mL and treated with supra-MIC concentrations of peptides. After 2 h treatment, bacterial cells were trapped by nitrocellulose membrane filters (0.1 μm CAS 900470.0 Ref. VCWP0/300) and then were fixed with 2% glutaraldehyde for 1 h. One percent of 1% osmium tetroxide (OsO_4_) was used as postfixation in 0.1 M sodium cacodylate buffer for 1 h. Samples were washed three times with cacodylate buffer (0.1 M sodium cacodylate) for 10 min at room temperature (RT). The samples were dehydrated with a graded ethanol series (50, 70, 96, and 100% alcohol). After the dehydration step, samples were dried by hexamethyldisilazane for 1 h and mounted on the specimen stubs and sputter coated with gold. An SEM-FEG (field emission guns) microscope (JEOL JSM 6700F) with an accelerating voltage of 30 kV was used.

### Cross-section TEM

*Escherichia* *coli* at the end-exponential growth phase were washed twice and diluted with physiological water and subsequently treated with either MIC value of specific aggregator peptides or buffer for 2 h (Control group) at 37 °C. After 2 h, bacterial cells were centrifuged at 4,000 × *g* for 4 min and pellets were fixed by 2.5% glutaraldehyde in 0.1 M Na-cacodylate buffer, pH = 7.2–7.4 (+2.5 mM CaCl_2_ + 1 mM MgCl_2_), for 1 h. Then, the pellets were washed with cacodylate buffer, re-suspended in 1.5% low melting point agarose (Sigma A4018) in cacodylate buffer (40 °C), and centrifuged at 4,000 × *g* for 4 min. The centrifuge tubes were placed on ice for 15 min, after which the tips containing the pellets were cut-off and the pellets were removed in a drop of cacodylate buffer. Pellets were cut into 1 mm³ cubes (4 °C), post-fixed with 1% OsO_4_ in distilled water for 2 h, and washed twice with distilled water. Thereupon, the samples were dehydrated in a graded ethanol series (30, 50, 70, 90, and 100%) for 5 min in each step at 4 °C while slowly rotating (ethanol 100%, repeated three times). Finally, cells were treated with propylene oxide (Sigma CAS number: 75-56-9) twice for 15 min at 4 °C, infiltrated with a 1:1 mixture of epoxy resin and propylene oxide (60 min at 4 °C, slowly rotating), and subsequently left in a mixture of 2:1 epoxy resin and propylene oxide overnight under a fume hood without caps. The next morning, samples were placed in 100% fresh epoxy resin, embedded in BEEM capsules in the evening, and polymerized for 2 days in an oven at 60 °C. Ultrathin sections were cut with a Leica ultracut UCT ultramicrotome and observed in a JEOL JEM-1400 TEM operated at 80 kV and equipped with an Olympus Quemesa 11Mpxl camera.

### In vitro hemolytic activity test

The hemolytic activities of peptides were determined by hemolysis against human erythrocytes. Pooled fresh blood was obtained from healthy volunteers (Red Cross Flanders) and erythrocytes were collected by centrifugation 1,000 × *g* for 5 min (anticoagulated by EDTAK). The pellet was washed three times with phosphate-buffered saline (PBS) and was diluted to a concentration of 8% in PBS. One hundred microliters of 8% red blood cells solution was mixed with 100 µL of serial dilutions of peptides in PBS buffer in 96-well plates (BD Biosciences). The reaction mixtures were incubated for 1 h at 37 °C. Thereupon, the plate was centrifuged for 10 min at 1,000 × *g* and 100 µL of supernatant was transferred to a sterilized 96-well plate (BD Biosciences, flat bottom). The release of hemoglobin was determined by measuring the absorbance of the supernatant at 405 nm. The hemolytic activity was determined as the minimal peptide concentration that caused hemolysis (minimal hemolytic concentration). Erythrocytes in 1% Triton and maximum used concentration of vehicle were used as the control of 100 and 0% hemolysis, respectively.

### In vitro mammalian cytotoxicity

Mammalian cytotoxicity was measured using the LDH release (Roche, Mannheim, Germany) and CellTiter Blue (Promega) methods. Briefly, HeLa cells (the human cervix epitheloid carcinoma cell line HeLa was obtained from the European Collection of Authenticated Cell Cultures (ECACC 93021013)) were seeded in 96-well round bottom plates at a concentration of 3 × 10^5^ cells/mL in Dulbecco’s modified Eagle’s medium and treated by different concentrations of peptides. Cells treated with 1% Triton™ X-100 and vehicle were considered as positive and negative controls, respectively. Microplates were incubated at 37 °C with 5% CO_2_ and 90% humidity for 4 h. The micro-plate was centrifuged at 200 × *g* for 10 min. One hundred microliters of supernatant was transferred to a clear 96-well flat bottom microplates. In order to determine LDH activity in the supernatants, 100 µl reaction mix (catalyst and dye solution) was added to each well and incubated for 30 min at RT in the dark and the LDH reaction was stopped by adding 100 µL of the stop solution. The absorbance of the samples was measured at 490 nm. The cell viability was calculated using the formula: (exp.value − negative control value)/(positive control value − negative control value)×100. The amount of absorbance is proportional to the number of living cells and corresponds to the cells’ metabolic activity. Cell lines were regularly tested for *Mycoplasma* contamination.

### Expression of HcaB in *E. coli* ATCC 25922 strain

The coding regions of *E*. *coli* O157 HcaB were amplified using *hcaB*-specific primers (5′-ATGTCGACATGAGCGATCTGCATAACGA-3′ and 5′-ATGTCGACATGGAGCGATTTATCGAAGAAGGC-3′, 5′-ATCCCGGGTTAAAGATCCAACCCAGCCG-34′) containing *Sal*I and *Sma*I restriction sites for cloning purposes. Two truncate versions of the gene, one with the targeted gene part, APR, and the other one without the target region were designed. For genomic DNA  (gDNA) of bacteria *E*. *coli* O157 strain, a clinical isolate was used as a template. The amplicons were ligated into *Sal*I/*Sma*I-digested pCN68 *E*. *coli*– Staphylococcus  shuttle vectors yielding different truncated version of pCN-hcaB. In this plasmid, PblaZ is the promoter. Ampicillin (25 μg/mL) or erythromycin (100 μg/mL) was used as the selection markers. Correctness of cloning was confirmed first by restriction enzyme digestion, colony PCR, and nucleotide sequence analysis of the insert and then by sequencing.

### Knockout and co-expression chaperone strains

Knockout chaperon strains were purchased from Dharmacon *E*. *coli* Keio knockout collection, which contains a set of single-gene deletion mutants for chaperone *E*. *coli* K-12 genes. Each deleted gene has been replaced with a kanamycin (30 μg/ mL) resistance cassette.

Chaperone co-expression plasmids were purchased from Takara set (catalog# 3340), which consists of five different plasmids that contain chaperone plasmids developed by HSP Research Institute Inc. Plasmids were transformed into competent *E*. *coli* BL21. The co-expression chaperone strains are chloramphenicol selective of chaperone plasmid (20 μg/mL), and chaperone inducer depending on the chaperone plasmid used. pG-KJE8 (DnaK-dnaJ-grpE araB groES-groEL) induced by l-arabinose (0.5 mg/mL), tetracycline (1–5 ng/mL), pGro7 (groES-groEL) induced by l-arabinose (0.5 mg/mL), pKJE7 (dnaK-dnaJ-grpE) induced by l-arabinose (0.5 mg/mL), pG-Tf2 (groES-groEL-tig) induced by tetracycline (1–5 ng/mL), and pTf16 (tig) induced by l-arabinose (0.5 mg/mL).

### Macrolide and peptide interaction

To evaluate the effect of peptides in the presence of erythromycin, *E*. *coli* O157 was grown in 5 mL of LB. Exponential-phase cultures were then diluted to 10^8^ cells/mL. Bacterial cells were treated with erythromycin at the concentration of 100 μg/mL for 3 h to stop growing at 37 °C, without shaking. Different concentrations of peptides (from 100 to 0.75 μg/mL) or buffer were plated in 96-well plates with at least three replicate wells (50 μL). Fifty microliters of erythromycin-treated bacteria were added to each well and 96-well plate was incubated for 2 h at 37 °C. After 2 h, bacterial cells were serially diluted and cultured on blood agar plates. Plates were incubated at 37 °C overnight. The number of living cells was measured by CFU counting method.

### Fluorescence microscopy of co-cultures of bacteria and mammalian cell

For imaging purposes, human HeLa cells were grown on small-cell-view cellular dish with glass bottom (Greiner Bio-One GmbH/35 mm Ref: 627860) to form a confluent monolayer. Thereupon, cells were infected with 200 μL of an overnight culture of *E*. *coli* O157 strain with FITC peptide (3 × MIC) for 24 h. Cells were stained with CellMask Deep Red plasma membrane dye (Thermo Fisher catalog# C10046) and 1 μL of NucBlue reagent for 30 min (Invitrogen), and then the medium was removed and 2 mL paraformaldehyde 4% was added to the plate for fixation. The plate was incubated for 6 h at RT. The co-cultured cells were washed at least three times with 1 mL PBS prior to imaging.

### Staining with luminescent conjugated oligomers

Two hundred microliters of end-exponential culture *E*. *coli* O157 were washed with PBS for three times, the bacterial numbers were adjusted to 10^8^ cells, and afterwards, bacteria were treated with peptides (at MIC) or buffer for 1 to 2 h. After 2 h, cells were treated with LCO dye (pFTAA; 0.5 µM, ThT; 25 µM) for 90 min. The absorption and emission spectra were measured from 480 to 600 nm with excitation at 440 nm (20 nm bandpass).

### Flow cytometry analysis of bacteria treated by peptides

Using a double staining technique with propidium iodide (PI) and FITC peptides, killing rate and peptide uptake were evaluated in a two-dimensional analysis. Briefly, end-exponential growth phase *E*. *coli* O157 cells (10^8^ CFU/mL) were washed with PBS and treated with peptides (P2 or Pro2/ FITC labeled) at MIC value for different time periods. Treated bacteria were again washed with PBS buffer three times. One microliter of PI (Invitrogen) was added to the bacteria, and after incubation for 5 min, the mixture was aliquoted (500 µL) into FACS tubes. To correlate the activity of the peptides with cell death, the fluorescence intensity was measured in two channels using the Gallios^TM^ Flow Cytometer, PI: excitation 536 nm and emission 617 nm, FITC: excitation 490 nm and emission 525 nm. Heated bacteria at 90 °C for 10 min were used as PI-positive control.

### IB purification

Twenty milliliters of overnight culture of bacteria was centrifuged for 30 min at 4,000 × *g* and washed by physiological water. Bacterial cells were treated by peptide at MIC for half killing time; afterwards, the bacterial pellets were washed with 10 mL buffer A (50 mM HEPES, pH 7.5, 300 mM NaCl, 5 mM β-mercaptoethanol, 1.0 mM EDTA) and centrifuged at 4 °C for 30 min at 4,000 × *g*. The supernatant was discarded and 20 mL of buffer B (buffer A plus 1 μg/mL leupeptin, 0.1 mg/mL AEBSF (4-(2-aminoethyl)benzenesulfonyl fluoride hydrochloride)) was added to the bacterial pellet. In order to break the cells, a Glen Creston Cell Homogenizer with pressure set to 20,000–25,000 psi was used, and in addition, the suspensions were sonicated (Branson Digital sonifier 50/60 HZ) on ice with alternating 2 min cycle (15 pulses at 50% power with 30 s pauses on ice, until completing 2 min total sonication time). The lysed cells were centrifuged at 4 °C for 30 min at 11,000 × *g*. The precipitated fraction was afterward re-suspended with 10 mL buffer D (buffer A plus: 0.8% (V/V) Triton X-100, 0.1% sodium deoxycholate) and the suspension was sonicated to ensure the pellet is completely dissolved. This step was repeated three times. Centrifugation was performed at 4 °C for 30 min at 11,000 × *g*. Finally, to solubilize IB, the pellet was suspended in 1 mL of buffer F (50 mM HEPES, pH 7.5, 8.0 M urea) per gram of precipitated fraction.

### Peptide activity and stability in the presence of serum

Briefly, the blood was allowed to clot for 90 min at RT. The blood was then centrifuged at 1,000 × *g* for 10 min and serum was separated. Serum was diluted into RPMI medium Gibco™ (25 or 50%) and peptides with different concentration of 5, 25, and 50 μg/mL were added to each well. After 2 h incubation, end-exponential *E*. *coli* O157 culture was washed three times by PBS (7,100 × *g*, 10 min). The number of bacteria was then adjusted to 9 × 10^8^ cells in RPMI medium with or without serum. After 2 h incubation, bacterial cells were serially diluted and were cultured on blood agar plates. The plates were then incubated at 37 °C overnight. The number of living bacteria was quantified as the number of CFU/mL.

### HcaB purification and antibody production

The coding whole *E*. *coli hcaB* gene (SE2232, Taxonomy ID: 83333) was amplified using *hcaB*-specific primer (*5′-CATATG*ATG**CATCATCACCATCACCAC**AGCGATCTGCATAACGA-3′ and *5′-CCTAGG*TTAAAGATCCAACCCAGCCG-3′) with additional *Nde*I and *Bam*HI restriction site (underlined and italic) for cloning purposes. Anti-6xHis (bold) tag used as a tag on the recombinant proteins to facilitate protein purification.

gDNA of *E*. *coli* strain O157 was used as a template. The amplicon was ligated into *Nde*I/*Bam*HI-digested pET11C plasmid as a component of a system for protein expression in *E*. *coli* yielding pET-his-*hcaB*. In this plasmid, T7 RNA polymerase (highly active constitutive promoter) was the promoter. Ampicillin was used as the selection marker. All recombinant plasmids were replicated in *E*. *coli* BL21 to have BL21 pET-his-*hcaB*. The correctness of cloning was confirmed by restriction enzyme digestion, and nucleotide sequence analysis of the insert.

Protein was purified as described previously (Luminy and Cedex 2011). Briefly, *E*. *coli* BL21 pET-his-*hcaB* was grown in 1 L of LB broth with 100 μg/mL ampicillin and 1 mM isopropyl-β-d-thiogalactopyranoside (IPTG) at 37 °C overnight with shaking. The cells were harvested (1,700 × *g*, 10 min, 4 °C) and re-suspended in 25 mL lysis buffer (PBS, pH 7.5, 1 mM β-mercaptoethanol plus 1 tablet protease inhibitor Mini, EDTA-freelyse). Bacterial cells were lysed using French pressure cell press (EmulsiFlex-C3 homogenizer) and stirred at 4 °C by adding DnaseI, and whole proteins were purified. To get rid of aggregation, we kept the proteins on ice and then filtered it. HcaB protein purification was performed by AKTA FPLC system, which is a fully automated liquid chromatography system, where HcaB protein was purified using HiPrepTM HP 5 mL column. Before actual purification run, the AKTA were stripped, charged, and blanked. The purification was done by program in AKTA Xpress. The purified proteins were collected from different tubes and the combined fractions were kept at 4 °C. Purified proteins were checked by running on the SDS gel.

In order to make polyclonal antibodies, several Swiss mice were immunized twice IP, in 10 days. The first injection was administered with a mixture of HcaB protein (50 μg per mouse) and complete Freund’s adjuvant (Sigma) (1:1). On day 10, a booster injection (protein and incomplete Freund’s adjuvant (Sigma)) was given after titration of the antibody by enzyme-linked immunosorbent assay. Then from the serum, the total immunoglobulin Gs purified by absorption to a protein G column (GE Healthcare) according to the manufacturer’s instructions.

### Experimental animals

Female Swiss mice of 5 to 8 weeks with uniform weight (20 and 23 g) were used in this study (Harlan, The Netherlands). Mice were housed in plastic cages, five mice per cage on softwood granules as bedding. The room was kept between 21 °C and 25 °C with 12/12 h light–dark cycles. The animals had free access to water and pelleted rodent food. In order to avoid stress-induced confounding factors mice were transferred to the lab 1 week before experimental manipulation.

### In vivo toxicity test

The procedures used in the assays were approved by the local Animal Ethics Committee and conform to international standards of animal welfare (Approval P067/2015 of the Ethical Committee of KULeuven). A safe concentration of peptides of 30 mg/g was used for this experiment. The selected dose achieved from escalation experiment had no acute adverse effects upon IP administration.

Briefly, 5–6-week-old Swiss females were divided into three groups (six animals per group) and administered 30 mg/kg of P2 (group A) or vehicle (physiological water pH 7.5) (group B) once a day for 18 days via IP injection. During the treatment period, the clinical, physiological, and behavioral parameters, including body weight, food and water consumption, body condition score, home cage activity, and locomotion were constantly monitored and recorded.

Three days after the last administration, animals were anesthetized and blood was collected using a standard retro-orbital puncture from each animal. Next, the mice sacrificed and underwent complete necropsy with gross examination and organ weights. The organs (heart, liver, spleen, kidney, bone marrow, brain, lung) were sampled and immersed in fixative in 4% paraformaldehyde. Formalin-fixed tissues after dehydration were routinely processed and embedded in paraffin blocks for histopathological examination. Five-micrometer-thick sections taken from these blocks (Thermo Scientific Microm HM355S microtome) were then stained with hematoxylin and eosin (Leica ST5010 Autostainer XL) and evaluated under a Leica DM 2500 light microscope by a board-certified veterinary pathologist. Hematology examination was performed using an automated high-resolution flow cytometer, Abbott Cell-Dyn 3700.

### The urinary tract infection model

Eight-week-old Swiss female mice were used for the urinary tract infection model as described previously^39^. Briefly, mice were anesthetized by IP administration of Nembutal 10% and then with fingers the bladder of the mouse was massaged and pushed down gently on to expel remaining urine. Thereafter, the anesthetized mice were inoculated UT with 50 µL of bacterial suspension slowly (1 × 10^8^ CFU/mL) by the sterile catheter in the bladder over 5 s in order to avoid vesicoureteral reflux through a surgical microscope. We estimated the sample size as follows: allowing a type I error rate of 0.05, a type II error 0.2, and estimating the maximum standard deviation of the CFU determination at 1 log CFU; we calculated that a sample size of 15 would allow us to reliably detect an effect size of 1 log CFU difference between treated and untreated. After 1 h, mice were randomized and divided into five groups (15 mice per groups): groups A and B received 10 mg/kg P2 IP or UT, respectively, group C received P2Pro2 peptides (proline substitutions) via UT injection; group D received ampicillin orally as the positive control, and group E received the vehicle (physiological water). The catheter was then removed directly after inoculation. After surgery, the animals were visually monitored for full recovery. Twenty-four hours post infection, mice were sacrificed and organs (kidney, bladder, ureter, colon) were washed with PBS and homogenized (Thermo Savant FastPrep FP120 Homogenizer/24 s). The homogenized tissues were serially diluted and were cultured on blood agar plates. The plates incubated overnight at 37 °C and the rate of bacteria was measured by CFU value.

Blinding: Sample preparation and treatment of the animals was performed by La.d.K., L.al.K., who also performed the CFU determination; however, between treatment and readout, the animals were randomly shuffled by F.C. and the key to the grouping was not revealed until after all the results were received. No animals were excluded. For the ANOVA analysis of the infection model (Fig. 2f–i), the assumption that the groups have similar standard deviations was tested using Bartlett’s test, which showed no significant differences between the standard deviations, except for the ureter data, which we ignored because it was caused by a reduction in the standard deviation of the untreated group

### Construction of *E. coli* MG1655 *dnaK-mCer3*

To construct *E*. *coli* MG1655 *dnaK-mCer3*, plasmid pGBKD-*mCer3* was first constructed by integrating a *mCer3* amplicon, generated with primer pairs 5′-AGAATTCGGCAGCGGCAGCGGCAGCGTGAGCAAGGGCGAGGA-3′ (Fw) and 5′-AGGATCCTTACTTGTACAGCTCGTCCA-3′ (Rev), into pGBKDparSpMT1^40^ using *Eco*RI and *Bam*HI restriction sites. In addition to adding the respective restriction sites to the end of the amplicon, these primer pairs also add a flexible linker (encoding GSGSGS^41^) facilitating folding of fluorescent fusion proteins constructed with these sequences. A *mCer3-frt-cat-frt* cassette was subsequently PCR amplified from plasmid pGBKD-*mCer3* using primer pairs

5′-AGATGACGATGTTGTCGACGCTGAATTTGAAGAAGTCAAAGACAAAAAAGGCAGCG GCAGCGGCA-3′ (Fw) and 5′-AGGAAATTCCCCTTCGCCCGTGTC AGTATAATTACCCGTTTATAGGGCGA GTGTAGGCTGGAGCTGCTTC-3′ (Rev).

The amplicon was subsequently inserted into MG1655, creating a C-terminal DnaK-mCer3 fusion. The *cat* cassette was subsequently flipped out by transiently equipping this strain with plasmid pCP20^42^, resulting in the desired MG1655 *dnaK-mCer3* strain.

### Protein purification for MS analysis

For protein purification from SDS gel, the purified IBs were loaded on SDS gel (4–15% Mini-PROTEAN^®^ TGX™ Precast Protein Gels, 10-well, 50 µl well volume) and stained by Coomassie blue (R250). The excised bands were cut by a sterile scalpel under a laminar flow. The gel slices washed in several cycles by incubating them in 50 mM ammonium bicarbonate/acetonitrile (ACN) (1:1) for 10 min at RT until the blue stain is gone and replacing the buffer by 100% ACN and incubating for 5 min. After the last cycle, the samples were dried by Speedvac and digested with 250 ng of modified trypsin (Promega) in 50 mM ammonium bicarbonate buffer (pH 8.3) overnight at 37 °C. Peptides were extracted by adding 5% ACN + 0.1% formic acid, and followed by 10% ACN + 0.1% formic acid (FA) and 95% ACN in 0.5% FA and dried by Speedvac. The extracted peptides were cleaned up by using pierce C18 spin columns (Thermo Fisher Scientific) according to the manufacturer’s instructions. The samples were diluted in 10 µL with 5% ACN + 0.1% FA for injection in the Q Exactive Orbitrap mass spectrometer (Thermo Fisher Scientific, USA).

For gel-free mass spectrometry experiment, dithiothreitol (DTT) solution was added to purified IBs (final conc. of DTT 0.020 M) and incubated for 15 min at RT. IAA was added to the solution (final working conc. 0.050 M) and incubated for 30 min in the dark. ABC (final conc. 0.11 M) was added to samples with trypsin (0.2 μg trypsin to 20 μg protein). To ensure complete protein proteolysis, we allowed trypsin digestion to proceed for at least 16 h at 37 °C. Thereafter, peptides were cleaned up using C18 spin Columns (Thermo Fisher Scientific). According to the manufacturer’s instructions, the samples were diluted in 10 µL with 5% ACN + 0.1% FA for injection in the Q Exactive Orbitrap mass spectrometer (Thermo Scientific, USA).

### Analysis of mass spectrometry data

Samples (5 µL) were digested and injected for UPLC separation using an Ultimate 3000 UPLC System (Dionex, Thermo Scientific), using an Acclaim PepMap100 pre-column (C18 3 μm–100 Å, Thermo Scientific) and a C18 PepMap RSLC (2 µm, 50 µm–15 cm, Thermo Scientific) using a linear gradient (300 µL/min) of 0–4% buffer B (80% ACN, 0.08% FA) for 3 min, 4–10% B for 12 min, 10–35% for 20 min, 35–65% for 5 min, 65–95% for 1 min, 95% for 10 min, 95–5% for 1 min, and 5% for 10 min. The Q Exactive Orbitrap mass spectrometer (Thermo Scientific, USA) was operated in positive ion mode with a nano spray voltage of 1.5 kV and a source temperature of 250 °C. Proteo Mass LTQ/FT-Hybrid ESI Pos. Mode Cal Mix (MS CAL5-1EASUPELCO, Sigma-Aldrich) was used as an external calibrant and the lock mass 445.12003 as an internal calibrant. The instrument was operated in data-dependent acquisition mode with a survey MS scan at a resolution of 70,000 (fw hm at *m*/*z* 200) for the mass range of *m*/*z* 400–1,600 for precursor ions, followed by MS/MS scans of the top 10 most intense peaks with +2, +3 +4, and +5 charged ions above a threshold ion count of 16,000 at 17,500 resolution using normalized collision energy of 25 eV with an isolation window of *m*/*z*3.0 and dynamic exclusion of 10 s. All data were acquired with Xcalibur 3.0.63 software (Thermo Fisher Scientific). For identification, all raw data were converted into mgf.files by Proteome Discover version 1.4 (Thermo Fisher Scientific) and processed using MASCOT version 2.2.06 (Matrix Science) against the Uniprot *E*. *coli* database. The parameters used to search at MASCOT were: parent tolerance of 10 ppm, fragment tolerance of 0.02 Da, variable modification oxidation of M, fixed modification with carbamidomethyl C, and up to one missed cleavage for trypsin. Results from Mascot were imported to Scaffold version 3.6.3. The parameters used in Scaffold for protein identification were to retain proteins with 99% confidence and containing at least two identified peptides with confidence level 95%.

### Nano-FTIR

For mid-IR near-field spectroscopy, a commercial scattering-type scanning near-field optical microscopy system was used (Neaspec GmbH, Germany). The system is based on standard AFM technology where a conventional metal-coated (Pt/Ir) tip is vertically oscillating. The tip acts simultaneously as an AFM probe and as a near-field probe^43^. Tip and sample were illuminated via a parabolic mirror objective with a broadband mid-infrared supercontinuum laser (Neaspec GmbH, max frequency range ca. 650–2,100 cm^−1^, the average power of 1 mW), which is generated by difference frequency generation. The tip-scattered light is analyzed with an asymmetric Fourier transform spectrometer where tip and sample are located in one of the interferometer arms. The detector signal is demodulated at a frequency O2 (2x AFM tip oscillation frequency) for effective background suppression. An interferogram is measured by recording the demodulated detector signal as a function of the position of the reference mirror at a fixed tip position. Subsequent Fourier transform of the recorded interferogram yields the complex-valued near-field point spectrum. The complex spectrum can be represented as nano-FTIR reflectivity and absorption spectra^44,45^.

### Statistical methods

All experiments were performed in minimum three replicates. For statistical evaluation of the determined averages and standard deviations of the mean, data were analyzed for significant differences using the statistical tests indicated in the figure legends or text, and included corrections for multiple testing when required. Statistical calculations were performed using Prism or R, unless otherwise indicated. Asterisks indicating the level of the *P* value centered over the error bar mean: *P < 0.05, **P < 0.01, ***P < 0.001, and ****P < 0.0001.

### Data availability

Data supporting the findings of this manuscript are available from the corresponding authors upon reasonable request. The mass spectrometry proteomics data have been deposited to the ProteomeXchange Consortium via the PRIDE^46^ partner repository with the accession codes PXD008685 and 10.6019/PXD008685, as well as PXD008701 and 10.6019/PXD008701.

## Electronic supplementary material

Supplementary Information

Description of Additional Supplementary Files

Supplementary Data 1

Supplementary Data 2

Supplementary Data 3
