# Supplementary material for: Aggregating sequences that occur in many proteins constitute weak spots of bacterial proteostasis
Source: Nat Commun. 2018 Feb 28;9:866. doi: 10.1038/s41467-018-03131-0 (PMC5830399; doi:10.1038/s41467-018-03131-0)
Supplement: Supplementary file 1 — Supplementary Information [file 41467_2018_3131_MOESM1_ESM.docx]

**Supplementary Figure 1 -** Distribution of APR sequence redundancy in bacterial proteomes: percentage identical sequences (red), 1 mismatch (blue) and 2 mismatches (green). (A) *Klebsiella pneumoniae*, (B) *Pseudomonas aeruginosa,* (C) *Acinetobacter baumannii*.

**Supplementary Figure 2 - Biophysical characterization of P2. (A)** ESI–IMS–MS Driftscope plot of the P2 monomer (1) through to the nonamer (9) present two minutes after diluting the monomer to a final peptide concentration of 100 μM in 50 mM MES buffer pH 7. ESI–IMS–MS Driftscope plots show the IMS drift time versus mass/charge (*m/z*) versus intensity (z, square-root scale). Data are from a single experiment that was replicated 3 times. **(B)** Histogram of the size distribution of particles calculated from DLS data recorded of P2 0, 1.5 and 3 h after dissolving the peptide using a linear polymer as particle model. **(C)** Solubility of P2 (black bars) and P2Pro (grey bars) peptide samples 30min, 18h and 42h after dissolving upon ultracentrifugation at 250,000 g for 2h (average and SD of 4 replicates). **(D)** FTIR spectrum of the insoluble fractions obtained after 30min in the previous panel (average and SD of 4 replicates). **(E)** Time-dependence of the fluorescence intensity of the amyloid-specific dye pFTAA added to preparation of P2, P2Pro or vehicle control in the presence of 1 mM hexaphosphate (polyP). (average and SD of 4 replicates). **(F)** Fluorescence emission spectra of pFTAA added to P2 or P2Pro in the presence or absence of 1 mM hexaphosphate (polyP). **(G)** Fluorescence emission spectra of Thioflavin T (ThT) added to P2 or P2Pro in the presence or absence of polyphosphate (polyP). **(H)** and **(I)** Representative transmission electron microscopy images of P2 (negative staining with uranyl acetate).

**Supplementary Figure 3 – SIM images of pFTAA staining of *E. coli* BL21 treated with toxic and non-toxic peptides (25 µg mL**^-1^**).** The peptides used are indicated on the panels.

**Supplementary Figure 4 - Structural characterization of inclusion bodies.** **(A)** Fluorescence emission spectrum of the amyloid specific dye pFTAA in *E. coli* O157: H7 treated with buffer, P2Pro and P2 (average of 3 repeats). **(B)** Fluorescence intensity of *E. coli* O157: H7 or *E. coli* BL21, stained with Thioflavin T after 2h treatment with P2 or P2Pro (average and SD of 3 measurements) **(C-E)** Nano-FTIR analysis of cross-sections of *E. coli* O157: H7 treated with P2 embedded in resin. Sample topography **(C)**, coupled with AFM phase imaging **(D)**, reveals nanometer-scale sample roughness. **(E)** Near-field amplitude image (integrated intensity) provides similar contrast for inclusion bodies as observed in electron microscopy. **(F)** Nano-FTIR spectra (1400-1800 cm^-1^), colour-coded as the box areas marked in E. Spectra obtained from inclusions indicate a prevalence for β-sheet structure, as revealed by analysis of the amide I and II regions, in contrast to random measurements obtained from areas within the *E. coli* cytoplasm. Background reference spectra were also obtained, revealing no contributions in the amide I region for β-sheet components. (Statistical significance is indicated as follows: * P ≤ 0.05, ** P ≤ 0.01, *** P ≤ 0.001, **** P ≤ 0.0001)

**Supplementary Figure 5.** Scanning electron microscopy (SEM) of *E. coli* strains, *E. coli* BL21 (A-G) or *E. coli* O157: H7 (H-J). Conditions were: **(A)** treated with 100 µg mL^-1^ of Ampicillin for 120 min at 37 °C. **(B)** untreated bacteria at 37 °C. **(C)** treated with 50 µg mL^-1^ of the bee-venom derived peptide melittin for 120 min at 37 °C. **(D)** BL21 overexpressing the DNA binding domain (DBD) of human p53, expression was induced with 0.4 mM of Isopropyl β-D-1-thiogalactopyranoside (IPTG) for 120 min at 37 °C. **(E)** Treated with 100 µg mL^-1^ of the proline-variant of P2 for 120 min at 37 °C. **(F)** Treated with P2 at MIC for 30 min at 37 °C. **(G)** Same as F, but treatment for 120 min **(H)** Mock treated bacteria (physiological buffer) for 120 min at 37 °C. **(I)** Treated with P105 at 4xMIC for 120 min at 37 °C. **(J)** Treated with P14 at 4X MIC for 120 min at 37 °C.

**Supplementary Figure 6 –** Western blot using the p53-specific monoclonal antibody pAb240 of recombinantly purified p53CD, of the inclusion body (IB) fraction of *E. coli* BL21 cells overexpressing p53CD and of wild type *E. coli* BL21.

**Supplementary Figure 7 -** Coomassie-blue-stained SDS-PAGE gel following separation of inclusion bodies purified from *E. coli* BL21 (A) or O157: H7 (B) after treatment with the peptides indicates or overexpression of p53CD. (C) and (D) show the average and standard deviation of the quantifications of the total intensity per lane from three biological replicates of the gels such as shown in (A) and (B), normalized on each gel to the untreated control. For BL21 statistically different samples were detected using ANOVA, followed by Tukey post-hoc comparison of all the samples to P2Pro. This shows that untreated, P4 and P53T samples are similar to P2Pro, whereas the lethal peptides and p53CD expression stand out. This type of analysis failed for the O157: H7 strain due to the higher variability observed, the overall pattern is however very similar.

**
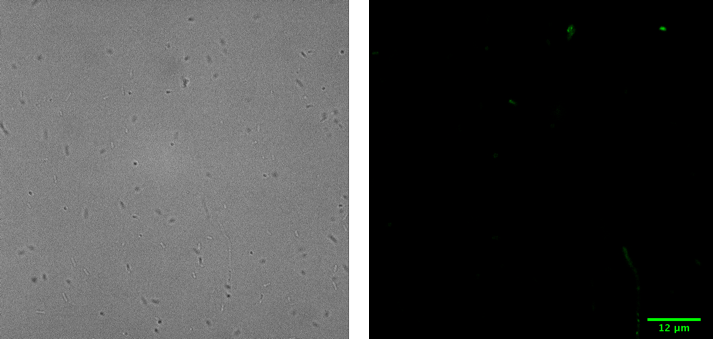
**

**Supplementary Figure 8 –** Structured Ilumination Miscroscopy (SIM) images of *E. coli* treated with p53T (right) and bright field images of the same field (left).

**Supplementary Figure 9 –** Fluorescence Activated Cell Sorting (FACS) of *E. coli* O157: H7 treated with P2 in the presence and absence of the ribosome inhibitor erythromycin.

**
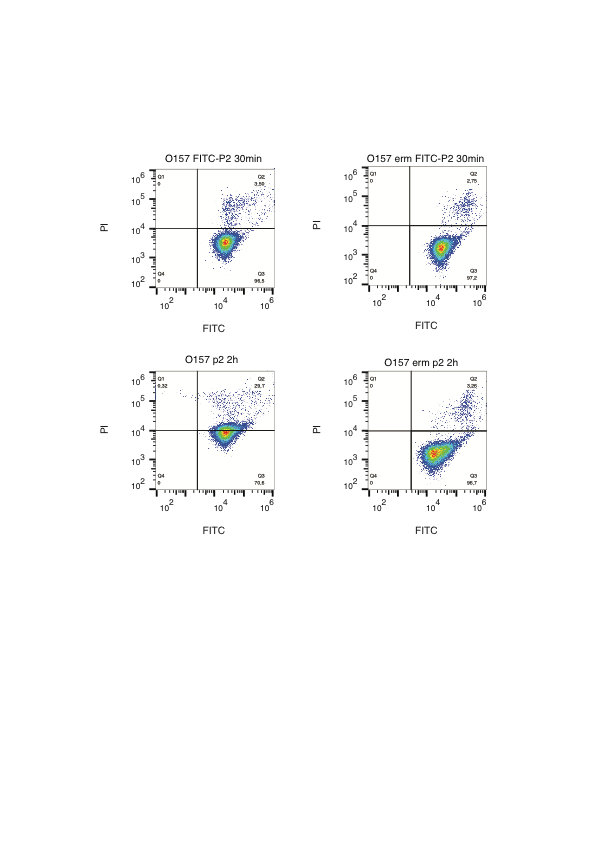
**

**Supplementary Figure 10 –** Fluorescence Activated Cell Sorting (FACS) of *E. coli* O157: H7 treated with FITC-P2 in the presence and absence of the ribosome inhibitor erythromycin.

**
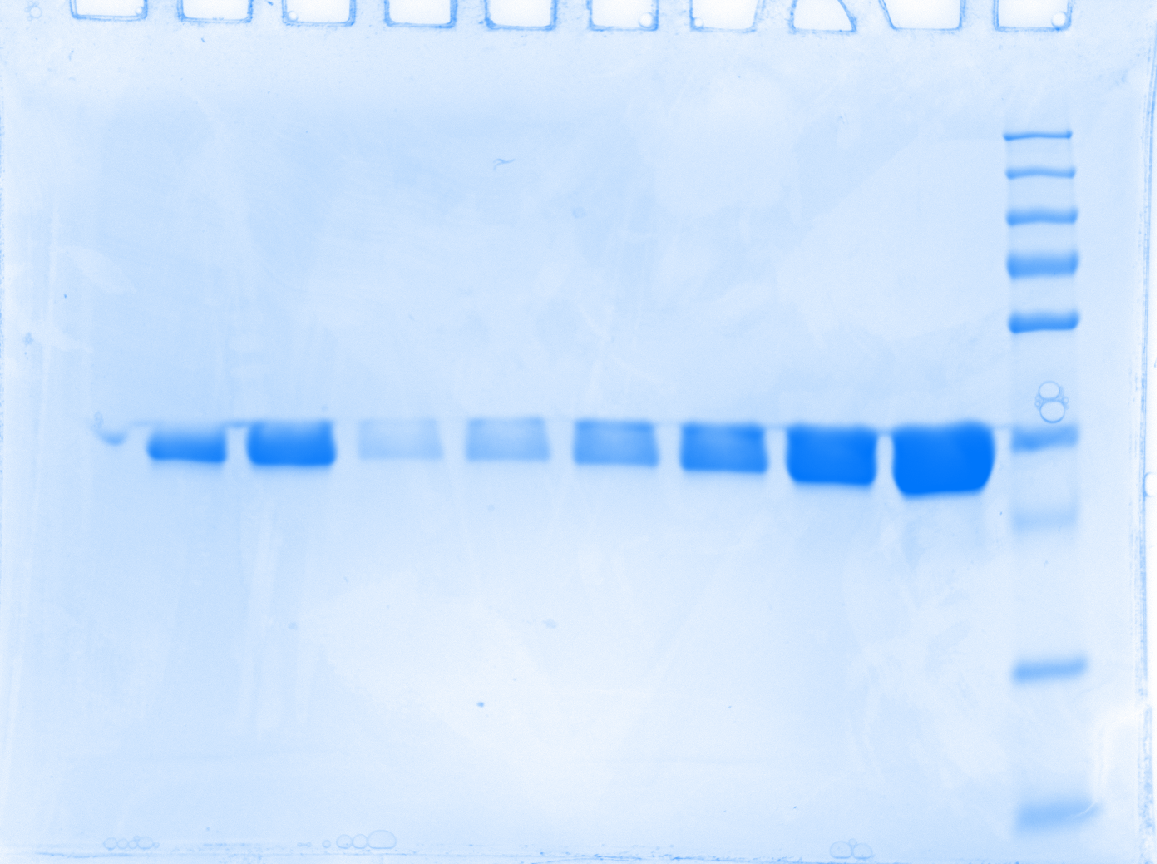
**

**Supplementary Figure 11 -** Coomassie gel of purified recombinantly HcaB.

**Supplementary Figure 12 – IB characterisation. (A)** Western blot of the soluble and insoluble fraction of *E. coli* BL21 and O157: H7 bacteria treated with P2 using the antibody raised against recombinantly produced HcaB protein. **(B)** Western blot using anti-HcaB polyclonal of soluble and insoluble fractions of *E. coli* O157: H7 treated with P2 or P2Pro.

**Supplementary Figure 13 – Comparisons of proteins detected in IBs. (A)** Calculated translational efficiency (according to Tuller et al^1^) of proteins predicted to co-aggregate with P2, divided into those that are detected by mass spectrometry (hits, see I) and those that were not detected (not). Boxplots as in B. **(B)** Average protein abundance in the PaxDB database of the proteins with an APR match to P2 that could be detected in mass spectrometry and those that could not. Boxplots as in B. Statistical test was unpaired student-t test. The bottom and top of the box are always the first and third quartiles, and the band inside the box represents the median. The whiskers are drawn using Tukey's method and show the extreme values that fall within 1.5 times the interquartile range. (Statistical significance is indicated as follows: * P ≤ 0.05, ** P ≤ 0.01, *** P ≤ 0.001, **** P ≤ 0.0001)

**
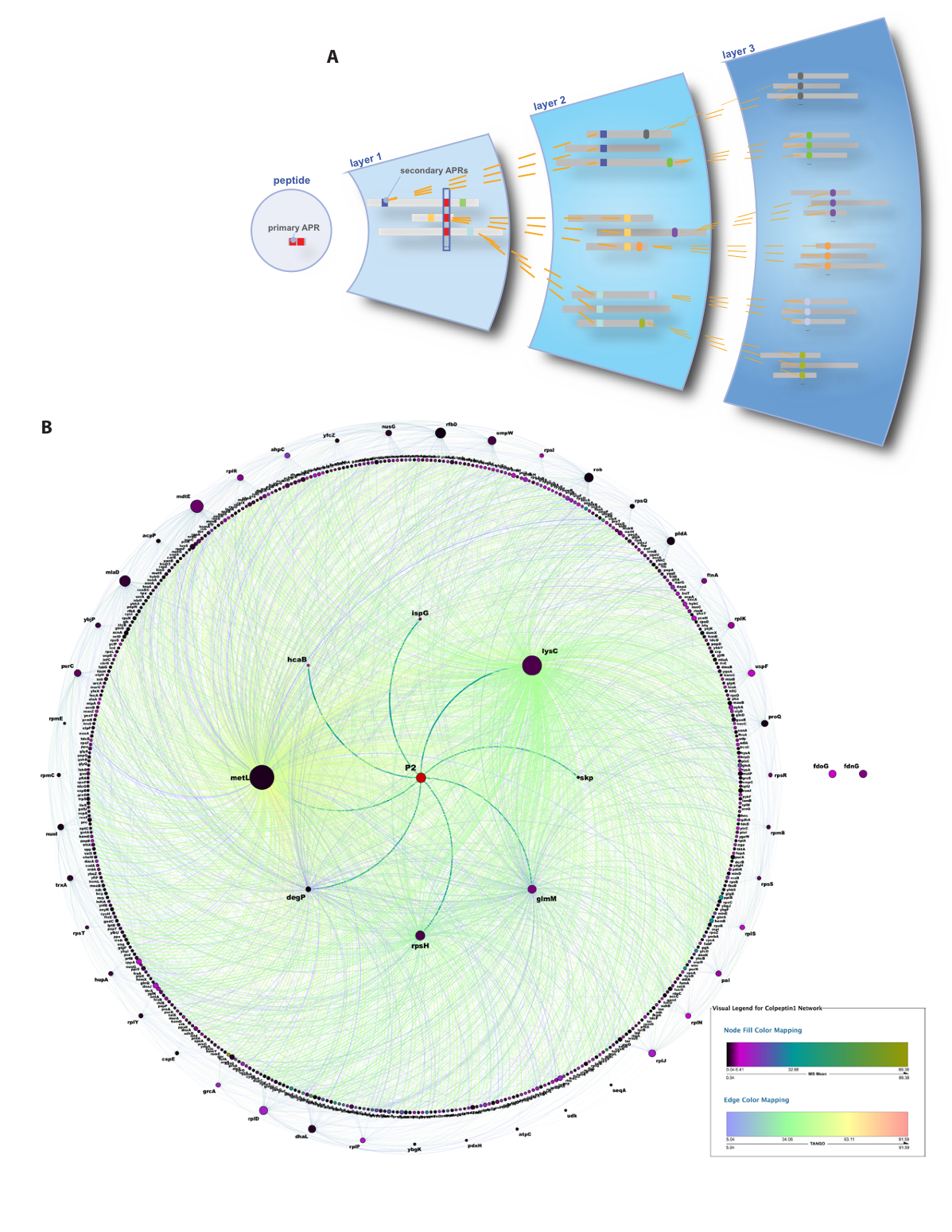
**

**Supplementary Figure 14. Co-aggregation network of P2 induced inclusion bodies. (A)** Schematic representing the co-aggregation cascade. The primary APR, which is encoded by P2, induces the aggregation of a set of proteins that share this sequence. These direct P2 targets in turn also contain other APRs that can be connected to a second layer of co-aggregation. By the third layer all but two proteins detected by the mass spectrometry are englobed in one co-aggregation network that is connected by nodes constituted of shared APRs. **(B)** Co-aggregation network derived from the mass spectrometry data of the P2 inclusion bodies. Edges are coloured by the TANGO score of the connecting APRs. Node colour gradient is based on signal intensity of the corresponding peptides in the mass spectrometry data. Node size is based on node degree. Edge thickness increases per layer level. Outliers are shown as single nodes on the right. Generated using Cytoscape^2^.

**Supplementary Figure 15 –** Correlation analysis of the mass spectrometry analysis of IB composition in *E. coli* (A) BL21 and (B) O157. The plots were generated using the corrplot package in R. The color scale represents the correlation value [-1, 1] and only correlation with a significance p-value < 0.01 were shown in the plot.

**Supplementary Figure 16 – Comparison of the Number of proteins found in IB in toxic and non-toxic conditions. (A)** Comparison of the total number of proteins identified in IBs of *E. coli* BL21 from bactericidal peptides (P2, P5, P14) versus non-lethal aggregating peptides (P2Pro, P4) and overexpression of p53CD. **(B)** Comparison of the specific number of proteins identified in addition to the IB-resident proteins in IBs of *E. coli* BL21 from bactericidal peptides (P2, P5, P14) versus non-lethal aggregating peptides (P2Pro, P4) and overexpression of p53CD. Statistical test was unpaired student-t test. (Statistical significance is indicated as follows: * P ≤ 0.05, ** P ≤ 0.01, *** P ≤ 0.001, **** P ≤ 0.0001)

**
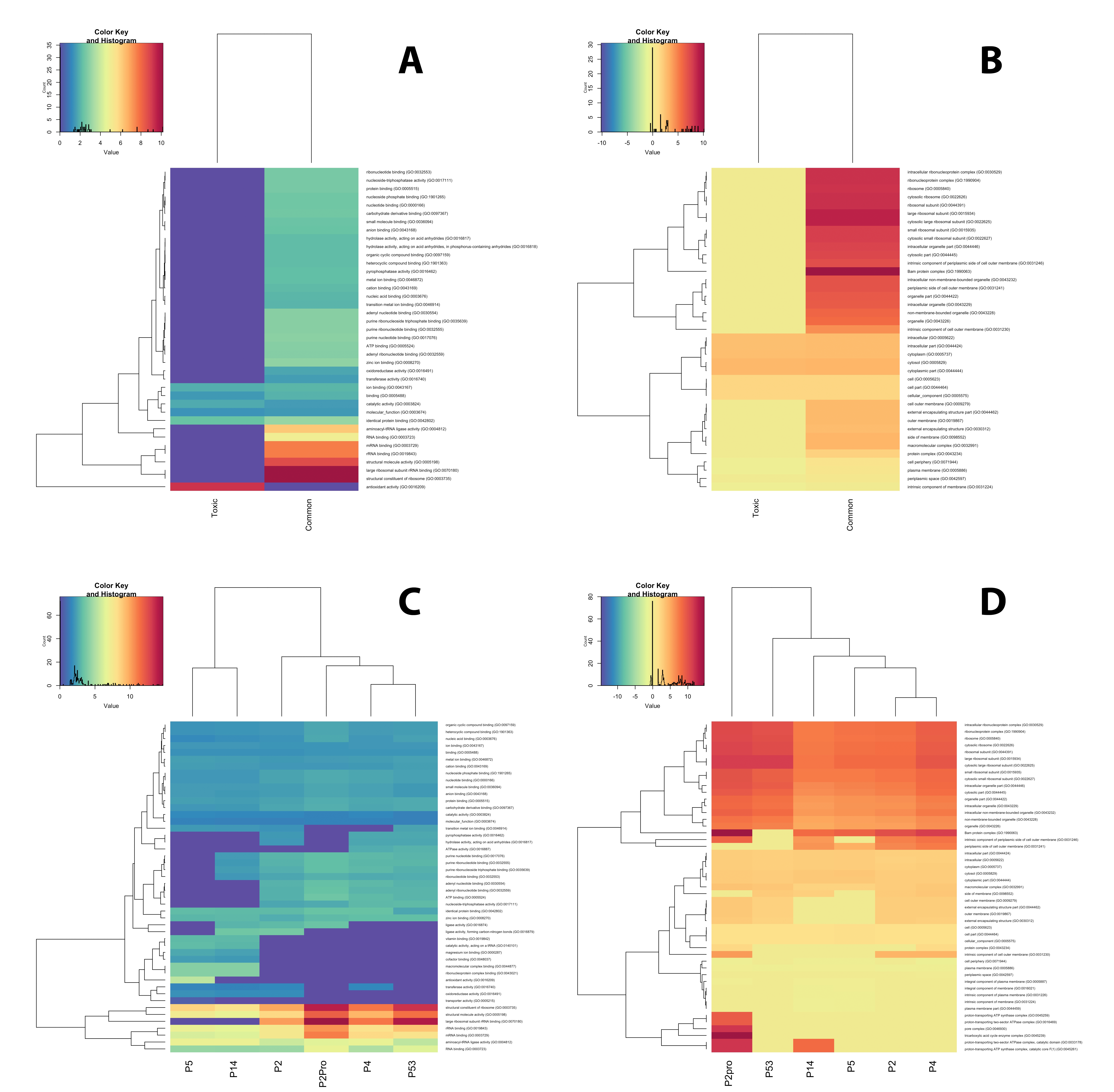
**

**Supplementary Figure 17 –** Gene ontology (GO) analysis heat maps of IB protein content. (A) GO analysis of the molecular functions and (B) cellular compartment of proteins located only in IBs of toxic peptides, compared to proteins constituting IBs of both toxic and non-toxic peptides. (C) Molecular functions and (D) cellular compartments of proteins located in IBs of cells treated with the different peptides. The color scale indicates the relative fold enrichment, the dendrograms show the relation between the samples. Histograms shown in black lines indicate the distribution of the enrichment values.

**
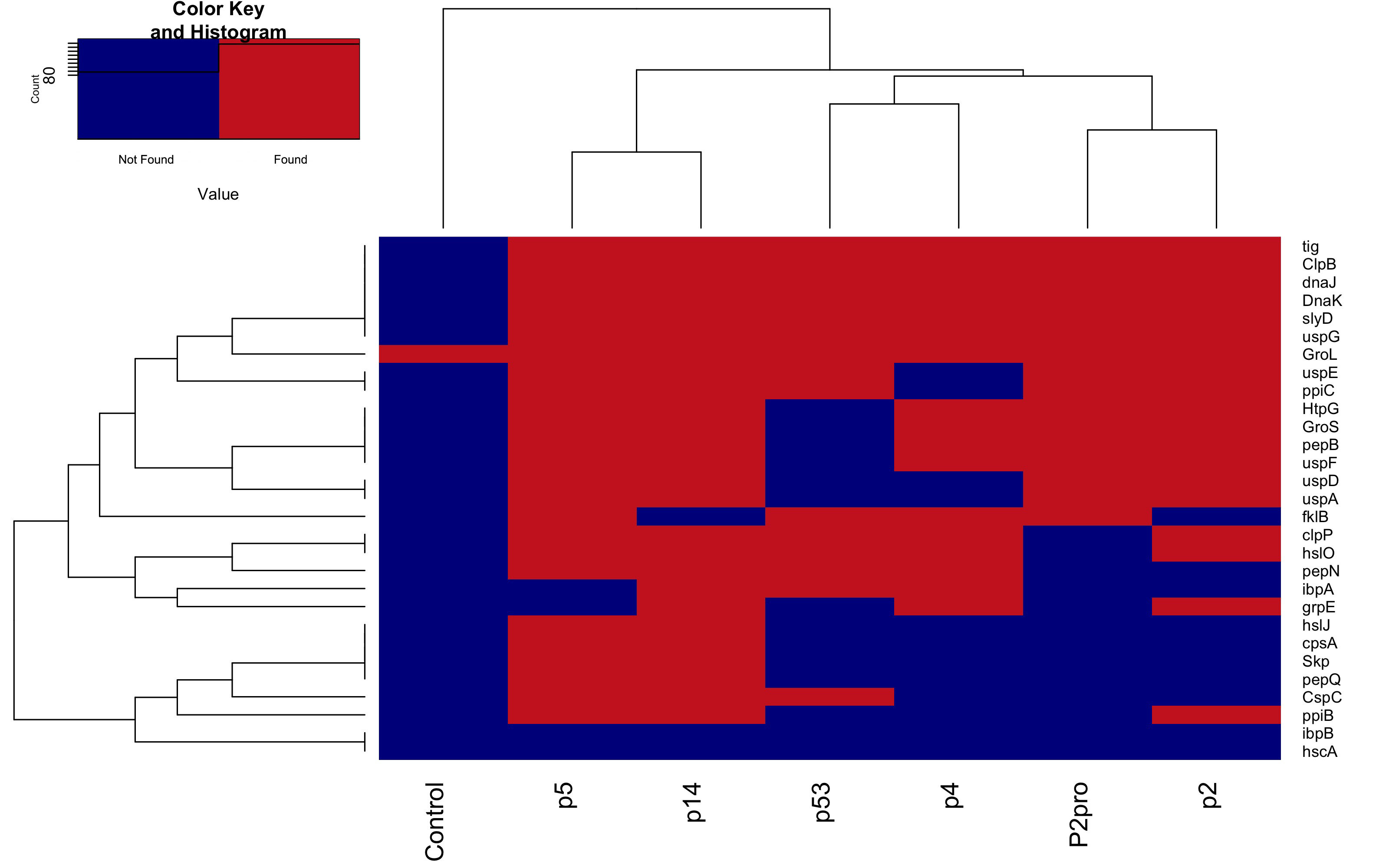
**

**Supplementary Figure 18** – Heat map of the molecular chaperones detected in the IB fraction of *E. coli* BL21 cells treated with different peptides or expressing p53CD. The color scale indicates the presence or absence of chaperons per sample, the dendrograms show the relation between the samples and between chaperones.

**
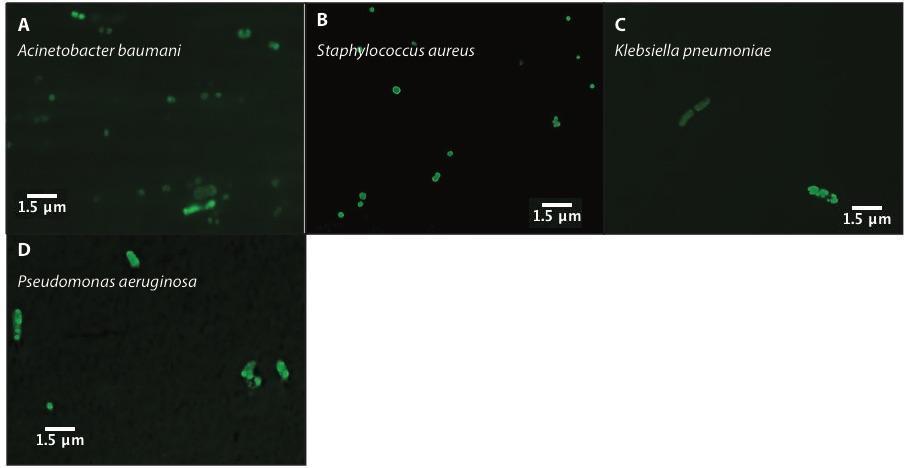
**

**Supplementary Figure 19 – Uptake of FITC-P2 in various bacterial strains, imaged using SIM microscopy.**

**Supplementary Figure 20 – Body weights of the treated mice by P2 for 18 days injection via IP.**

**
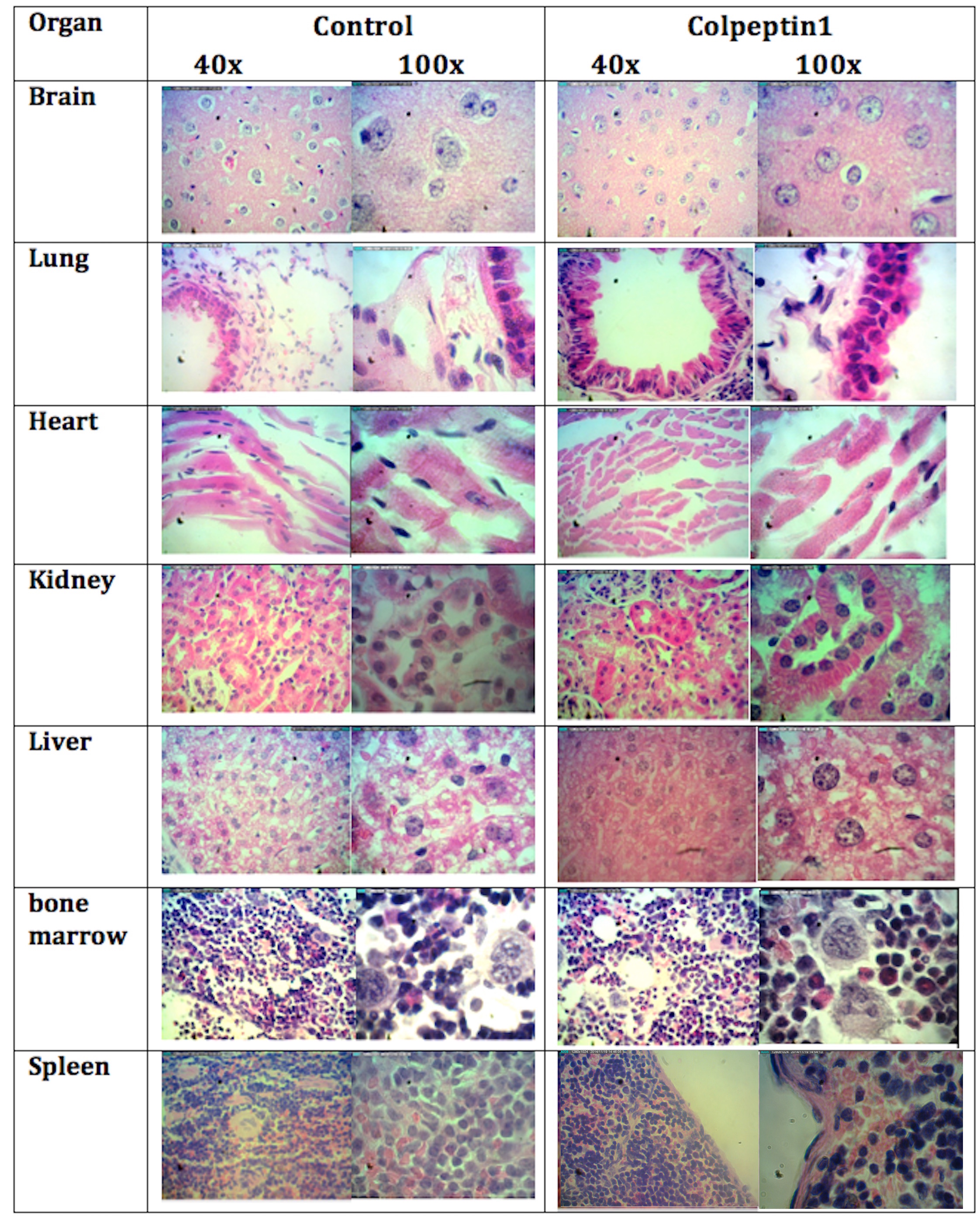
**

**Supplementary Figure 21** **– Pathological analysis of tissue sections of mice treated with 30 mg kg**^-1^ **P2 for 18 consecutive days.**

**
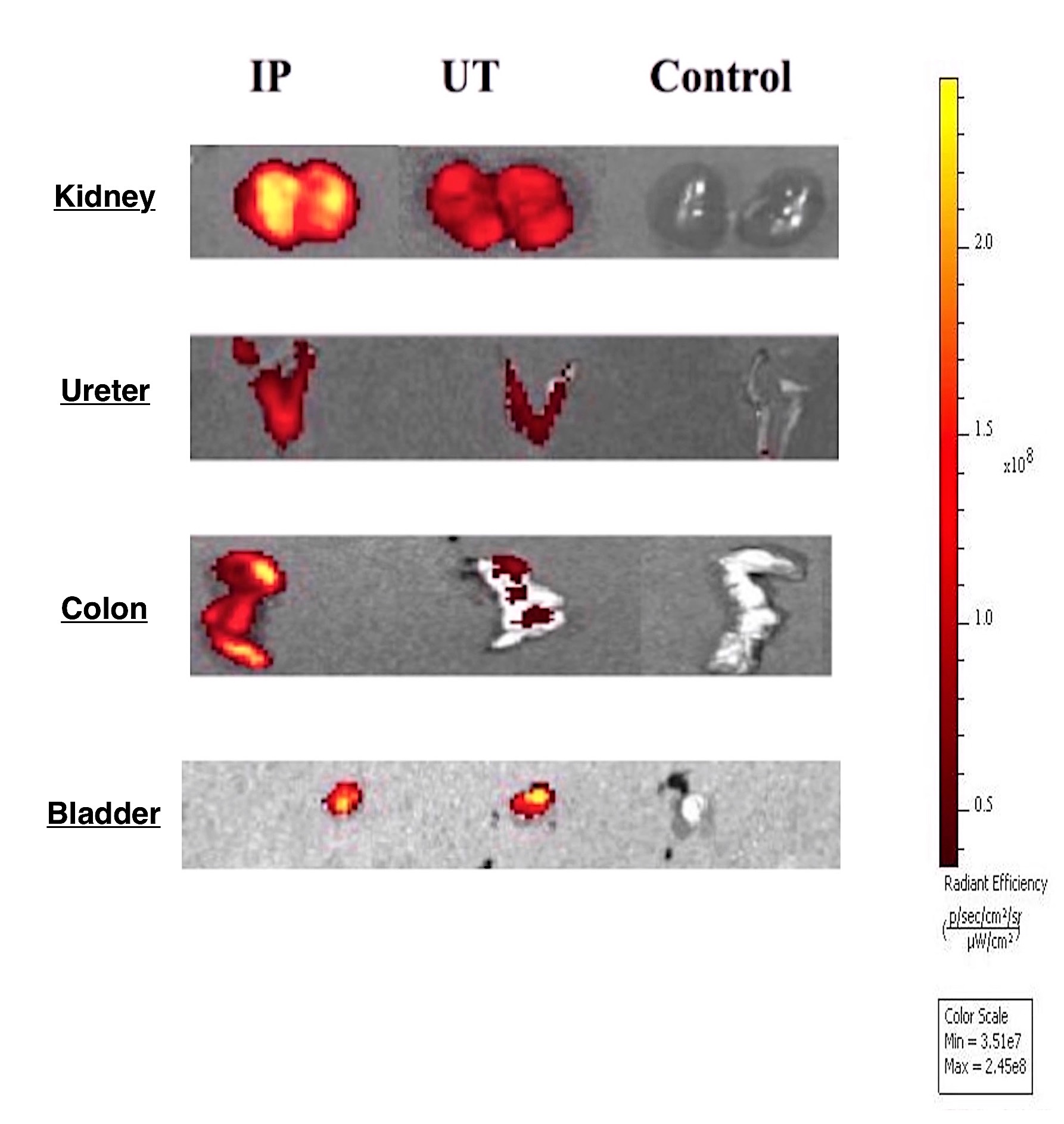
**

**Supplementary Figure 22 – Fluorescence imaging of organs *ex vivo* 3h after a single injection (10 mg kg**^-1^**) of P2. IP: Intraperitoneal injection, UT: Transurethral injection.**

**Supplementary Tables**

**Supplementary Table 1** – Peptide design and screening

| **Name** | **Polypeptide** | **APR** | **TANGO score** | **Matches^1^** | **APR length** | **peptide sequence** | **CAMP^2^** |
| --- | --- | --- | --- | --- | --- | --- | --- |
| P1 | EBESCP00000213293 | LLLSLLV | 76.83 | 22 | 7 | RLLLSLLVRRPRLLLSLLVRR | AMP |
| P2 | EBESCP00000210864 | GLGLALV | 20.85 | 19 | 7 | RGLGLALVRRPRGLGLALVRR | AMP |
| P3 | EBESCP00000209910 | LLLALLS | 57.27 | 19 | 7 | RLLLALLSRRPRLLLALLSRR | AMP |
| P4 | EBESCP00000209975 | LALALLL | 44 | 17 | 7 | RLALALLLRRPRLALALLLRR | AMP |
| P5 | EBESCP00000207772 | ALLTTLL | 20.71 | 16 | 7 | RALLTTLLRRPRALLTTLLRR | AMP |
| P6 | EBESCP00000212702 | TVTVTFG | 32.55 | 16 | 7 | RTVTVTFGRRPRTVTVTFGRR | NAMP |
| P7 | EBESCP00000212122 | TVTVTFG | 32.7 | 16 | 7 | RTVTVTFGRRPRTVTVTFGRR | NAMP |
| P8 | EBESCP00000209162 | IGALLLL | 39.24 | 15 | 7 | RIGALLLLRRPRIGALLLLRR | AMP |
| P9 | EBESCP00000212122 | TVTVTFN | 32.28 | 15 | 7 | RTVTVTFNRRPRTVTVTFNRR | NAMP |
| P10 | EBESCP00000209965 | ALIAALQ | 21.77 | 14 | 7 | RALIAALQRRPRALIAALQRR | AMP |
| P11 | EBESCP00000211133 | VLALAAL | 37.47 | 14 | 7 | RVLALAALRRPRVLALAALRR | AMP |
| P12 | EBESCP00000207824 | ALAVALL | 72.72 | 13 | 7 | RALAVALLRRPRALAVALLRR | AMP |
| P13 | EBESCP00000211390 | AVLGLLA | 41.53 | 13 | 7 | RAVLGLLARRPRAVLGLLARR | AMP |
| P14 | EBESCP00000208655 | GLLALLA | 33.3 | 13 | 7 | RGLLALLARRPRGLLALLARR | AMP |
| P15 | EBESCP00000213158 | LIGIALG | 33.04 | 13 | 7 | RLIGIALGRRPRLIGIALGRR | AMP |
| P16 | EBESCP00000210379 | ALLTAVL | 33.53 | 12 | 7 | RALLTAVLRRPRALLTAVLRR | AMP |
| P17 | EBESCP00000210098 | QLVALLV | 66.61 | 12 | 7 | RQLVALLVRRPRQLVALLVRR | AMP |
| P18 | EBESCP00000212988 | SAVLALL | 43.39 | 12 | 7 | RSAVLALLRRPRSAVLALLRR | AMP |
| P19 | EBESCP00000212122 | VVTVTLN | 50.97 | 12 | 7 | RVVTVTLNRRPRVVTVTLNRR | NAMP |
| P20 | EBESCP00000212555 | AVVLATG | 24.03 | 11 | 7 | RAVVLATGRRPRAVVLATGRR | AMP |
| P21 | EBESCP00000209874 | LLLIVLG | 81.63 | 11 | 7 | RLLLIVLGRRPRLLLIVLGRR | AMP |
| P22 | EBESCP00000213098 | ALAVAIG | 21.08 | 10 | 7 | RALAVAIGRRPRALAVAIGRR | AMP |
| P23 | EBESCP00000210425 | ALLITLL | 74.9 | 10 | 7 | RALLITLLRRPRALLITLLRR | AMP |
| P24 | EBESCP00000212549 | GLLLALQ | 30.59 | 10 | 7 | RGLLLALQRRPRGLLLALQRR | AMP |
| P25 | EBESCP00000212702 | IVTVTLN | 48.24 | 10 | 7 | RIVTVTLNRRPRIVTVTLNRR | AMP |
| P26 | EBESCP00000211310 | LFVGLAL | 39.45 | 10 | 7 | RLFVGLALRRPRLFVGLALRR | AMP |
| P27 | EBESCP00000212038 | VLGLAAL | 21.58 | 10 | 7 | RVLGLAALRRPRVLGLAALRR | AMP |
| P28 | EBESCP00000208389 | VVGLLAG | 29.48 | 10 | 7 | RVVGLLAGRRPRVVGLLAGRR | AMP |
| P29 | EBESCP00000211990 | ATVLALL | 25.16 | 9 | 7 | RATVLALLRRPRATVLALLRR | AMP |
| P30 | EBESCP00000212858 | AVLVAIG | 75.97 | 9 | 7 | RAVLVAIGRRPRAVLVAIGRR | AMP |
| P31 | EBESCP00000209426 | GLLVTLA | 36.24 | 9 | 7 | RGLLVTLARRPRGLLVTLARR | AMP |
| P32 | EBESCP00000209882 | LFVILAL | 76.02 | 9 | 7 | RLFVILALRRPRLFVILALRR | AMP |
| P33 | EBESCP00000211614 | LGIAVAL | 20.13 | 9 | 7 | RLGIAVALRRPRLGIAVALRR | AMP |
| P34 | EBESCP00000208407 | LLLLVNL | 68.83 | 9 | 7 | RLLLLVNLRRPRLLLLVNLRR | AMP |
| P35 | EBESCP00000212122 | TVTVALG | 25.08 | 9 | 7 | RTVTVALGRRPRTVTVALGRR | NAMP |
| P36 | EBESCP00000207816 | VGVIVGA | 42.18 | 9 | 7 | RVGVIVGARRPRVGVIVGARR | AMP |
| P37 | EBESCP00000212873 | VVVAIAL | 92.48 | 9 | 7 | RVVVAIALRRPRVVVAIALRR | AMP |
| P38 | EBESCP00000212079 | AGLLSLV | 24.67 | 8 | 7 | RAGLLSLVRRPRAGLLSLVRR | AMP |
| P39 | EBESCP00000210861 | ALLIQLL | 39.25 | 8 | 7 | RALLIQLLRRPRALLIQLLRR | AMP |
| P40 | EBESCP00000212018 | AQVLALL | 51.57 | 8 | 7 | RAQVLALLRRPRAQVLALLRR | AMP |
| P41 | EBESCP00000209188 | AVVLAVN | 72.36 | 8 | 7 | RAVVLAVNRRPRAVVLAVNRR | AMP |
| P42 | EBESCP00000210332 | FVAGFIG | 64.88 | 8 | 7 | RFVAGFIGRRPRFVAGFIGRR | AMP |
| P43 | EBESCP00000210737 | LAIALAQ | 25.46 | 8 | 7 | RLAIALAQRRPRLAIALAQRR | AMP |
| P44 | EBESCP00000212024 | LFIIATA | 66.7 | 8 | 7 | RLFIIATARRPRLFIIATARR | AMP |
| P45 | EBESCP00000209032 | LIVAAIA | 73.49 | 8 | 7 | RLIVAAIARRPRLIVAAIARR | AMP |
| P46 | EBESCP00000211255 | LLAGIVA | 34.55 | 8 | 7 | RLLAGIVARRPRLLAGIVARR | AMP |
| P47 | EBESCP00000209206 | LLLAYLL | 87.85 | 8 | 7 | RLLLAYLLRRPRLLLAYLLRR | AMP |
| P48 | EBESCP00000208308 | LLLMLAG | 50.96 | 8 | 7 | RLLLMLAGRRPRLLLMLAGRR | AMP |
| P49 | EBESCP00000212564 | LLTLLNL | 20.02 | 8 | 7 | RLLTLLNLRRPRLLTLLNLRR | AMP |
| P50 | EBESCP00000211085 | LVGLVLG | 46.09 | 8 | 7 | RLVGLVLGRRPRLVGLVLGRR | AMP |
| P51 | EBESCP00000210979 | LVVTAIA | 55.99 | 8 | 7 | RLVVTAIARRPRLVVTAIARR | AMP |
| P52 | EBESCP00000207741 | PVIILTA | 68.83 | 8 | 7 | RPVIILTARRPRPVIILTARR | AMP |
| P53 | EBESCP00000212005 | QAIVITG | 30.47 | 8 | 7 | RQAIVITGRRPRQAIVITGRR | AMP |
| P54 | EBESCP00000210487 | TVVLLAA | 57.57 | 8 | 7 | RTVVLLAARRPRTVVLLAARR | AMP |
| P55 | EBESCP00000209584 | AALITAL | 23.95 | 7 | 7 | RAALITALRRPRAALITALRR | AMP |
| P56 | EBESCP00000208677 | AALLAYV | 59.49 | 7 | 7 | RAALLAYVRRPRAALLAYVRR | AMP |
| P57 | EBESCP00000210682 | AITLVLT | 42.08 | 7 | 7 | RAITLVLTRRPRAITLVLTRR | AMP |
| P58 | EBESCP00000207808 | ALVSLLL | 34.45 | 7 | 7 | RALVSLLLRRPRALVSLLLRR | AMP |
| P59 | EBESCP00000211568 | GIVGLVG | 43.59 | 7 | 7 | RGIVGLVGRRPRGIVGLVGRR | AMP |
| P60 | EBESCP00000213181 | GLAVGVI | 49.09 | 7 | 7 | RGLAVGVIRRPRGLAVGVIRR | AMP |
| P61 | EBESCP00000208911 | GTVLLVS | 52.8 | 7 | 7 | RGTVLLVSRRPRGTVLLVSRR | AMP |
| P62 | EBESCP00000210871 | GVALVVA | 77.53 | 7 | 7 | RGVALVVARRPRGVALVVARR | AMP |
| P63 | EBESCP00000212804 | GVLAVFA | 78.55 | 7 | 7 | RGVLAVFARRPRGVLAVFARR | AMP |
| P64 | EBESCP00000208059 | ILLLTLV | 98.77 | 7 | 7 | RILLLTLVRRPRILLLTLVRR | AMP |
| P65 | EBESCP00000210221 | IVIVGGG | 27.51 | 7 | 7 | RIVIVGGGRRPRIVIVGGGRR | AMP |
| P66 | EBESCP00000211867 | LCLLLAL | 39.47 | 7 | 7 | RLCLLLALRRPRLCLLLALRR | AMP |
| P67 | EBESCP00000209059 | LLAILAS | 42.95 | 7 | 7 | RLLAILASRRPRLLAILASRR | AMP |
| P68 | EBESCP00000211824 | LLIAVGA | 54.41 | 7 | 7 | RLLIAVGARRPRLLIAVGARR | AMP |
| P69 | EBESCP00000210873 | LLIVLGA | 75.39 | 7 | 7 | RLLIVLGARRPRLLIVLGARR | AMP |
| P70 | EBESCP00000211959 | NVVLLAL | 68.93 | 7 | 7 | RNVVLLALRRPRNVVLLALRR | AMP |
| P71 | EBESCP00000210460 | PAIVAAV | 33.39 | 7 | 7 | RPAIVAAVRRPRPAIVAAVRR | AMP |
| P72 | EBESCP00000211391 | QLLLTLL | 73.1 | 7 | 7 | RQLLLTLLRRPRQLLLTLLRR | AMP |
| P73 | EBESCP00000209484 | SAIIGII | 60.99 | 7 | 7 | RSAIIGIIRRPRSAIIGIIRR | AMP |
| P74 | EBESCP00000212211 | VSLVAIL | 57.68 | 7 | 7 | RVSLVAILRRPRVSLVAILRR | AMP |
| P75 | EBESCP00000209467 | VVALVAG | 60.61 | 7 | 7 | RVVALVAGRRPRVVALVAGRR | AMP |
| P76 | same as P1 |  |  |  | 7 | RLRLSLLVRRPRLLLSLLVRR | AMP |
| P77 | same as P2 |  |  |  | 7 | RGLRLALVRRPRGLGLALVRR | AMP |
| P78 | same as P3 |  |  |  | 7 | RLLLARLSRRPRLLLALLSRR | AMP |
| P79 | same as P4 |  |  |  | 7 | RLALRLLLRRPRLALALLLRR | AMP |
| P80 | same as P5 |  |  |  | 7 | RALRTTLLRRPRALLTTLLRR | AMP |
| P81 | same as P6 |  |  |  | 7 | RTVTRTFGRRPRTVTVTFGRR | NAMP |
| P82 | same as P7 |  |  |  | 7 | RTVTVRFGRRPRTVTVTFGRR | NAMP |
| P83 | same as P8 |  |  |  | 7 | RIRALLLLRRPRIGALLLLRR | AMP |
| P84 | same as P9 |  |  |  | 7 | RTVTVTRNRRPRTVTVTFNRR | NAMP |
| P85 | same as P10 |  |  |  | 7 | RALRAALQRRPRALIAALQRR | AMP |
| P86 | same as P11 |  |  |  | 7 | RVLARAALRRPRVLALAALRR | AMP |
| P87 | same as P12 |  |  |  | 7 | RALRVALLRRPRALAVALLRR | AMP |
| P88 | same as P13 |  |  |  | 7 | RAVRGLLARRPRAVLGLLARR | AMP |
| P89 | same as P14 |  |  |  | 7 | RGLLARLARRPRGLLALLARR | AMP |
| P90 | same as P15 |  |  |  | 7 | RLRGIALGRRPRLIGIALGRR | AMP |
| P91 | same as P16 |  |  |  | 7 | RALLTARLRRPRALLTAVLRR | AMP |
| P92 | same as P17 |  |  |  | 7 | RQLVARLVRRPRQLVALLVRR | AMP |
| P93 | same as P18 |  |  |  | 7 | RSARLALLRRPRSAVLALLRR | AMP |
| P94 | same as P19 |  |  |  | 7 | RVVTVRLNRRPRVVTVTLNRR | AMP |
| P95 | same as P20 |  |  |  | 7 | RAVRLATGRRPRAVVLATGRR | AMP |
| P96 | same as P21 |  |  |  | 7 | RLRLIVLGRRPRLLLIVLGRR | AMP |
| P97 | same as P22 |  |  |  | 7 | RARAVAIGRRPRALAVAIGRR | AMP |
| P98 | same as P23 |  |  |  | 7 | RARLITLLRRPRALLITLLRR | AMP |
| P99 | same as P24 |  |  |  | 7 | RGLRLALQRRPRGLLLALQRR | AMP |
| P100 | same as P25 |  |  |  | 7 | RIVRVTLNRRPRIVTVTLNRR | AMP |
| P101 | same as P1 |  |  |  | 7 | RLLRSLLVRRPRLLLSLLVRR | AMP |
| P102 | same as P2 |  |  |  | 7 | RGLGRALVRRPRGLGLALVRR | AMP |
| P103 | same as P3 |  |  |  | 7 | RLLLALRSRRPRLLLALLSRR | AMP |
| P104 | same as P4 |  |  |  | 7 | RLALARLLRRPRLALALLLRR | AMP |
| P105 | same as P5 |  |  |  | 7 | RALLRTLLRRPRALLTTLLRR | AMP |
| P106 | same as P6 |  |  |  | 7 | RTVTVRFGRRPRTVTVTFGRR | NAMP |
| P107 | same as P7 |  |  |  | 7 | RTVTVTRGRRPRTVTVTFGRR | NAMP |
| P108 | same as P8 |  |  |  | 7 | RIGRLLLLRRPRIGALLLLRR | AMP |
| P109 | same as P9 |  |  |  | 7 | RTVTVTFRRRPRTVTVTFNRR | NAMP |
| P110 | same as P10 |  |  |  | 7 | RALIRALQRRPRALIAALQRR | AMP |
| P111 | same as P11 |  |  |  | 7 | RVLALRALRRPRVLALAALRR | AMP |
| P112 | same as P12 |  |  |  | 7 | RALARALLRRPRALAVALLRR | AMP |
| P113 | same as P13 |  |  |  | 7 | RAVLRLLARRPRAVLGLLARR | AMP |
| P114 | same as P14 |  |  |  | 7 | RGLLALRARRPRGLLALLARR | AMP |
| P115 | same as P15 |  |  |  | 7 | RLIRIALGRRPRLIGIALGRR | AMP |
| P116 | same as P16 |  |  |  | 7 | RALLTAVRRRPRALLTAVLRR | AMP |
| P117 | same as P17 |  |  |  | 7 | RQLVALRVRRPRQLVALLVRR | AMP |
| P118 | same as P18 |  |  |  | 7 | RSAVRALLRRPRSAVLALLRR | AMP |
| P119 | same as P19 |  |  |  | 7 | RVVTVTRNRRPRVVTVTLNRR | NAMP |
| P120 | same as P20 |  |  |  | 7 | RAVVRATGRRPRAVVLATGRR | AMP |
| P121 | same as P21 |  |  |  | 7 | RLLRIVLGRRPRLLLIVLGRR | AMP |
| P122 | same as P22 |  |  |  | 7 | RALRVAIGRRPRALAVAIGRR | AMP |
| P123 | same as P23 |  |  |  | 7 | RALRITLLRRPRALLITLLRR | AMP |
| P124 | same as P24 |  |  |  | 7 | RGLLRALQRRPRGLLLALQRR | AMP |
| P125 | same as P25 |  |  |  | 7 | RIVTRTLNRRPRIVTVTLNRR | AMP |

^1^ The number of matching sequences in the *E. coli* O157: H7 proteome, allowing 1 mutation.

^2^ Prediction of the antibacterial activity using the CAMP software^3^.

**Supplementary Table 2-** MIC of the active peptides against *E. coli* O157 under 25 μg mL^-1^ concentration.

| **Number** | **Name** | | ***E. coli* O157**  **(μg mL**^-1^**)** |
| --- | --- | --- | --- |
| 1 | P2 | 6 | |
| 2 | P3 | 12 | |
| 3 | P4 | 6 | |
| 4 | P5 | 12 | |
| 5 | P12 | 25 | |
| 6 | P14 | 12 | |
| 7 | P16 | 25 | |
| 8 | P18 | 12 | |
| 9 | P23 | 25 | |
| 10 | P26 | 25 | |
| 11 | P29 | 25 | |
| 12 | P33 | 25 | |
| 13 | P39 | 25 | |
| 14 | P40 | 25 | |
| 15 | P49 | 12 | |
| 16 | P50 | 6 | |
| 17 | P58 | 12 | |
| 18 | P72 | 25 | |
| 19 | P76 | 12 | |
| 20 | P79 | 12 | |
| 21 | P80 | 25 | |
| 22 | P87 | 25 | |
| 23 | P88 | 25 | |
| 24 | P89 | 12 | |
| 25 | P90 | 25 | |
| 26 | P91 | 25 | |
| 27 | P92 | 6 | |
| 28 | P93 | 25 | |
| 29 | P99 | 25 | |
| 30 | P101 | 12 | |
| 31 | P103 | 25 | |
| 32 | P105 | 6 | |
| 33 | P111 | 25 | |
| 34 | P112 | 25 | |
| 35 | P113 | 25 | |
| 36 | P114 | 25 | |
| 37 | P115 | 25 | |
| 38 | P116 | 6 | |
| 39 | P117 | 25 | |
| 40 | P118 | 25 | |
| 41 | P123 | 25 | |
| 42 | P124 | 12 | |
| 43 | P125 | 25 | |

**Supplementary Table 3** – Proteins from the *E. coli* proteome with a sequence segment similar to the APR of P2.

| **APR** | **% Sequence ID** | **Gene** | **UniProt** | **Protein Name** | **PaxDB abundance (ppm)** | **Solubility (%)** |
| --- | --- | --- | --- | --- | --- | --- |
| GLGLALV | 100 | hcaB | HCAB_ECOLI | 3-phenylpropionate-dihydrodiol/cinnamic acid-dihydrodiol dehydrogenase | 0.015 | 10 |
| GLGLALA | 85.71 | skp | SKP_ECOLI | Chaperone protein skp | 796 | 49 |
| GLGLAIV | 85.71 | phoR | PHOR_ECOLI | Phosphate regulon sensor protein PhoR | 13.3 | 13 |
| GLGLAMV | 85.71 | dtpA | DTPA_ECOLI | Dipeptide and tripeptide permease A | 8.42 | NA |
| GLGLSLV | 85.71 | yedV | YEDV_ECOLI | Probable sensor-like histidine kinase YedV | 3.44 | 13 |
| GLALALV | 85.71 | yjcE | YJCE_ECOLI | Uncharacterized Na(+)/H(+) exchanger YjcE | 2.04 | NA |
| GLGLAIV | 85.71 | envZ | ENVZ_ECOLI | Osmolarity sensor protein EnvZ | 1.99 | NA |
| GLGLAIV | 85.71 | rstB | RSTB_ECOLI | Sensor protein RstB | 1.25 | 54 |
| GLGLAVV | 85.71 | zraS | ZRAS_ECOLI | Sensor protein ZraS | 0.816 | NA |
| GLPLALV | 85.71 | ybfO | YBFO_ECOLI | Putative uncharacterized protein YbfO | 0.48 | 8 |
| GVGLALV | 85.71 | dcuS | DCUS_ECOLI | Sensor histidine kinase DcuS | 0.185 | 35 |
| GLGLALS | 85.71 | atoS | ATOS_ECOLI | Signal transduction histidine-protein kinase AtoS | 0.153 | 27 |
| GLLLALV | 85.71 | hycD | HYCD_ECOLI | Formate hydrogenlyase subunit 4 | 0.01 | NA |
| GLLLALV | 85.71 | yddG | YDDG_ECOLI | Aromatic amino acid exporter YddG | 0.01 | NA |
| GLGLALQ | 85.71 | yfcJ | YFCJ_ECOLI | UPF0226 protein YfcJ | 0.01 | NA |
| GIGLALV | 85.71 | yfeZ | YFEZ_ECOLI | Inner membrane protein yfeZ | 0.01 | 41 |
|  |  |  |  |  |  |  |

**Supplementary Table 4 -** Minimum Inhibitory Concentration of P2 against clinical isolates of *E. coli* and *A. baumannii* and their resistance profile against established antibiotics.

Abbreviations: Amoxi/Clav - Amoxicillin+clavulanate, Piper/tazo - Piperacillin/tazobactam, Trimet/Sulfameto - Trimetoprim-sulfametoxazol.

+/- indicates strains that were deemed sensitive/resistant to the treatment.

**Supplementary Table 5 –** Organs weights of mice treated with 30 mg kg^-1^ P2 after 18 consecutive days injection.

|  | **Organs weights** | **(gr)** |
| --- | --- | --- |
|  | **Control** | **P2** |
| **Heart** | 0,20±0,01 | 0,20±0,02 |
| **Kidneys** | 0,58±0,01 | 0,58±0,01 |
| **Spleen** | 0,12±0,01 | 0,12±0,01 |
| **Liver** | 1,75±0,11 | 1,90±0,11 |
| **Brain** | 0,60±0,03 | 0,56±0,02 |

**Supplementary Table 6** – Hematological values (mean+SD) of mice treated by P2 after 18 consecutive days of injection.

| **Hematological** | **Control** | **P2** |
| --- | --- | --- |
| **Parameters** | **Mean + SD** | **Mean + SD** |
| **WBC** | 5,63 ± 0,16 | 6,89 ± 0,42 |
| **NEU** | 0,49 ± 0,03 | 0,80 ± 0,06 |
| **LYM** | 4,70 ± 0,02 | 5,32 ± 0,52 |
| **MONO** | 0,099 ± 0,04 | 0,15 ± 0,05 |
| **EOS** | 0,058 ± 0,01 | 0,14 ± 0,06 |
| **BASO** | 0,270 ± 0,02 | 0,24 ± 0,02 |
| **RBC** | 10,02 ± 024 | 10,9 ± 0,46 |
| **HGB** | 19,60 ± 0,20 | 17,8 ± 0,45 |
| **HCT** | 99,43 ± 2,06 | 98,87 ± 3,7 |
| **MCV** | 280,4 ± 1,06 | 273 ± 1,32 |
| **MCH** | 49,3 ± 1,090 | 48,2 ± 0,34 |
| **MCHC** | 51,89 ± 0,70 | 53,7 ± 0,42 |
| **PLT** | 1997 ± 30,7 | 1595 ± 50,7 |
| **MPV** | 17,65 ± 0,09 | 16,36 ± 0,2 |
| **PCT** | 1,150 ± 0,06 | 0,88 ± 0,05 |
| **PDW** | 49,3 ± 0,11 | 49,9 ± 0,43 |

WBC,White Blood Cell or Leukocyte count. NEU, Neutrophil absolute count %N — Neutrophil percent. LYM, Lymphocyte absolute count %L — Lymphocyte percent. MONO, Monocyte absolute count %M — Monocyte percent. EOS, Eosinophil absolute count %E — Eosinophil percent. BASO, Basophil absolute count %B — Basophil percent. RBC, Red Blood Cell or Erythrocyte count. HGB, Hemoglobin concentration. HCT, Hematocrit. MCV, Mean Corpuscular Volume. MCH, Mean Corpuscular Hemoglobin. MCHC, Mean Corpuscular Hemoglobin Concentration. PLT, Platelet or Thrombocyte count. MPV, Mean Platelet Volume. PDW, Platelet Distribution Width. PCT, Plateletcrit.

**Supplementary Table 7 – Primer sequences.**

| Primer | Sequence | Restriction site | Tm° | GC % |
| --- | --- | --- | --- | --- |
| hcaB_Fw1 | ATGTCGACATGAGCGATCTGCATAACGA | SalI | 59.9 | 46% |
| hcaB_Fw2 | ATGTCGACATGGAGCGATTTATCGAAGAAGGC | SalI | 63.1 | 47% |
| hcaB_Rv | ATCCCGGGTTAAAGATCCAACCCAGCCG | SmaI | 64.3 | 57% |
| hcaB Fw_Purification | CATATGATGCATCATCACCATCACCACAGCGATCTGCATAACGA | NdeI | 68.3 | 45% |
| hcaB Rv_Purification | CCTAGGTTAAAGATCCAACCCAGCCG | BamHI | 61.1 | 54% |
| pGBKD-mCer3_Fw | AGAATTCGGCAGCGGCAGCGGCAGCGTGAGCAAGGGCGAGGA | EcoRI | 76.2 | 67% |
| pGBKD-mCer3_Rv | AGGATCCTTACTTGTACAGCTCGTCCA | BamHI | 59.7 | 48% |
| mCer3-frt-cat-frt _Fw | AGATGACGATGTTGTCGACGCTGAATTTGAAGAAGTCAAAGACAAAAAAGGCAGCGGCAGCGGCA | x | 74.1 | 48% |
| mCer3-frt-cat-frt _Rv | AGGAAATTCCCCTTCGCCCGTGTCAGTATAATTACCCGTTTATAGGGCGAGTGTAGGCTGGAGCTGCTTC | x | 76.4 | 51% |

**Supplementary Notes**

**Supplementary Note 1: P2 forms soluble oligomeric β-structured aggregates that mature into amorphous aggregates**

We characterized the *in vitro* aggregation behavior of P2 in more detail. Due to technical limitations, this has to be done in simplified aqueous buffers that are a poor mimic of the intracellular environment, and thus only show that the peptide can form aggregates in biocompatible physicochemical conditions, in line with the intended design, but give no guarantee that similar structures are formed in complex biological matrices. Electrospray ionisation-mass spectrometry linked to ion mobility spectrometry (ESI-IMS-MS)^4^ revealed that immediately following solubilisation of P2 in 100 mM ammonium acetate buffer the peptide is not only monomeric, but also readily forms soluble oligomers ranging from dimers up to 9-mers, and likely higher order (Supplementary Figure 2A). Consistent with this, the main species observed by Dynamic Light Scattering (DLS) upon dissolving have apparent hydrodynamic radii of approximately 1-2 nm, which quickly grow to large particles within a few hours (Supplementary Figure 2B). The composition of the P2 solution evolves over time towards larger species, which in the mass spectrometer is paralleled by a consumption of the smaller species (the larger being outside the detection range of the instrument). A study of the solubility of the peptide over time using ultracentrifugation also shows aggregation, with less than 60% of the peptide remaining in solution by ultracentrifugation 30 min after dissolving (Supplementary Figure 2C). The insoluble fraction collected in this manner was brought back into suspension (in 10% of the original volume) and the secondary structure content was analyzed using Fourier Transform Infrared Spectroscopy (FTIR), which shows major peaks around 1622 and 1641 cm^-1^, consistent with beta-structure formation (Supplementary Figure 2D). Upon exposure of P2 to polyphosphate (polyP), a naturally abundant form of phosphate in *E. coli* that was previously shown to facilitate amyloid formation ^5^, the peptide displays typical amyloid-aggregation kinetics as measured using the fluorescence intensity increase of p-FTAA (Supplementary Figure 2E). The pFTAA fluorescence emission spectrum showed the typical double maximum associated with amyloid formation (Supplementary Figure 2F). Peptide aggregates obtained in this manner were also positive for Thioflavin-T (Supplementary Figure 2G). By transmission electron microscopy mature aggregates mainly formed amyloid-like aggregates, which occasionally assembled into ordered fibrils (Supplementary Figure 2H & I). Analysis of the proline substituted and inactive control, P2Pro, showed that upon solubilisation under identical conditions, P2Pro is almost completely soluble and does not adopt a β-structured conformation, although sedimentation analysis still reveals aggregation on a longer timescale (Supplementary Figure 2C&D). Taken together, these features are consistent with P2 forming β-structured soluble oligomers that can rapidly convert to insoluble fiber-like aggregates, depending on conditions.

**Supplementary Note 2: Confirmation of cross-beta structure in peptide-induced IBs.** As mentioned in the main text the P2-induced IBs are positive for pFTAA (Supplementary Figure 4A), but to provide additional confirmation of the cross-beta nature of the proteins in the IBs, we also stained them with Thioflavin-T (Supplementary Figure 4B), the most widely used amyloid-sensor dye to date. To provide an orthogonal method, we resorted to the novel technique of correlative atomic force microscopy (AFM) nanoimaging and Fourier Transform Infrared Spectroscopy (FTIR) of the cross-sections of treated bacteria (Supplementary Figure 4C-F), as prepared for cross-section TEM (Figure 1G, H, I). This technique allows to obtain *in situ* FTIR absorbance spectra of inclusion bodies in cross-sections of bacteria and compare these to other regions of the same cells at 10 nm resolution. This showed clear peaks in the amide I region around 1616 and 1630 cm^-1^, consistent with beta-structure formation. There is also a component at 1647 cm^-1^ consistent with alpha-helical structures, but we also observe a peak at the wavenumber in the background, suggesting the beta-sheet component is more specific (Supplementary Figure 4F).

**Supplementary Note 3: Morphological analysis using Scanning Electron Microscopy (SEM) of bacteria treated at supra-MIC.** Bacteria were treated for 2 h and controls treated with buffer, ampicillin or the bee-venom derived membrane-active peptide melittin (Supplementary Figure 5). Bacteria treated with the bactericidal aggregating peptides P2, P14 or P105 appeared shrunken but without apparent membrane deformations such as observed with melittin, like blebbing and leaking. This makes massive membrane disruption as the main mechanism of cell killing very unlikely.

**Supplementary Note 4: A tandem peptide based on an aggregation prone region of the core domain of the human p53 protein.** We generated a tandem peptide, which we called p53T, following the same design pattern that was used to generate the sequences of the main screen in Table 1. This time however, instead of using a bacterial APR sequence, we incorporated the one APR identified by TANGO in the sequence of p53CD (ILTIITL)^6^, and that was experimentally confirmed by us and others to be highly aggregation prone^7, 8, 9^. Since this APR does not have a counterpart in a bacterial protein it is not expected to cause aggregation and by extension should not be toxic to *E. coli*, in line with the results of p53CD overexpression. This is indeed what we observed when we treated *E. coli* O157: H7 and BL21 with these peptides: there was no bactericidal effect, with MIC values greater than 100 μg mL^-1^ on both O157: H7 and BL21:DE3 *E. coli* strains. Also, super-resolution microscopy imaging of treated cells stained with pFTAA showed no inclusion body formation (Supplementary Figure 8) and coomassie gels of the IB fraction showed similar levels of protein accumulation as the controls (Supplementary Figure 7).

**Supplementary Note 5: A putative co-aggregation network of P2.** The P2 APR can be found in 15 other *E. coli* proteins (Supplementary Table 4) with a single mismatch and 158 proteins with a double mismatch. The direct APR match for P2 is the G_17_LGLALV_24_ sequence from *hcaB* (3-phenylpropionate-dihydrodiol/cinnamic acid-dihydrodiol dehydrogenase), a non-essential enzyme in the aromatic compound metabolism that occurs at very low abundance (0.015 ppm according to PaxDB^10^). Taguchi and co-workers have previously determined the solubility of the entire *E. coli* proteome including *hcaB* using a cell-free translation system showing that *hcaB* is a highly aggregation prone protein that is highly dependent on GroEL/ES for folding^11^. We could detect the presence of *hcaB* in P2 IBs by mass spectrometry analysis. To confirm this result, we cloned *hcaB* into an inducible vector for recombinant expression and chromatographically purified the protein from lysates of *E. coli* BL21:DE3 cells overexpressing *hcaB* (Supplementary Figure 11). Using this material, we performed an immunization scheme in mouse (see materials and methods), yielding antiserum against *hcaB* in a Western blot, revealing a band at the right molecular weight, as well as 2 off-target bands (Supplementary Figure 12A). Using this antiserum, we compared the effect of P2 treatment (1h at MIC concentration) on the presence of *hcaB* in the soluble and insoluble fraction of *E. coli* O157: H7, endogenously expressing *hcaB*, and *E. coli* BL21 cells overexpressing *hcaB* (Supplementary Figure 12A & B), confirming the accumulation of *hcaB* in the inclusion body fraction of P2 treated cells. Finally, we cut out the bands stained with the antiserum and reconfirmed the presence of *hcaB* content by mass spectrometry. Together this validates the mass spectroscopic analysis of IBs and confirms that *hcaB* does aggregate upon treatment with P2. In order to determine whether P2 also induces the aggregation of other putative P2 targets, we checked for the presence of these proteins in IBs in the mass spectrometry data (Supplementary Table 3) and found an additional 7 other P2 targets in P2-induced IBs. When calculating the translational efficiency of these genes based on Tuller’s method^1^, which uses typical decoding times of individual codons, we found that the translational efficiency of the 8 detected P2 target proteins was significantly higher (student t test, p<0.001, Supplementary Figure 13A) than for undetected putative targets, suggesting that a high translation rate might facilitate P2 induced aggregation. In addition, we noted that the proteins detected in the aggregates had a high average cellular abundance in *E. coli* according to the PaxDB database^10^ (Supplementary Figure 13B), which suggest protein abundance is limiting the detecting of aggregating proteins by the mass spectrometry method or that high protein abundance is an important prerequisite for co-aggregation.

Since we hypothesized that APR redundancy drives this proteostatic collapse by a sequence-specific cascade of protein aggregation, we analysed the 8 primary P2 targets identified by mass spectrometry for the presence of additional APRs in their sequence and checked whether these APRs could be detected in the remaining 533 P2 specific proteins (Supplementary Figure 14A). In other words, we analyzed whether P2-induced co-aggregated proteins present in these IBs can be connected by an aggregation cascade determined by secondary APRs in validated P2 targets. Intriguingly, an additional 488 proteins can be included in this manner, while an additional 43 proteins can be connected in a similar manner by a third layer (Supplementary Figure 14B), leaving 2 proteins (0.4%) unconnected, specifically the two subunits of formate dehydrogenase.

We cannot at present determine if the order of events suggested by the co-aggregation network is indeed the order of events that occurs upon peptide treatment, but the network presented here has the value of demonstrating that a model of sequence-specific co-aggregation events is capable of rationalizing seemingly complex mass spectrometry data.

**Supplementary Note 6:** We analyzed IB fractions extracted from *E. coli* BL21 and O157 cells after treatment with selected bactericidal (P2, P5, P14) and inactive (P2Pro, P4) peptides and compared it to IBs formed after transient overexpression of p53CD. In contrast to the gel-slicing methods employed for P2 IBs, we compensated for the high sample number by resorting to a shot-gun approach without sample fractionation prior to the inline chromatography (Supplementary Table 5). To assess the impact of the different conditions, we only considered proteins confidently identified in at least two out of three replicates for a given condition, amounting to 703 and 673 proteins in total for strains O157 and BL21, respectively. To allow direct comparison with p53CD overexpression, we decided to focus on the BL21 strain, a choice that was further supported by correlation analysis, which indicated a much higher internal consistency within the biological replicates per sample for BL21 than for O157: H7 (Supplementary Figure 15). Even in baseline conditions, the pathogenic *E. coli* O157: H7 strain showed fluctuations in the number of proteins in the IB fraction, consistent with a certain degree of proteostatic stress, whereas this was not observed with ‘laboratory strain’ BL21, perhaps because it experiences less stress from the manipulations (Supplementary Table 5). This internal proteostatic consistency of the BL21 strain, compared to O157:H7, is evident by the number of proteins found in their respective IBs in baseline conditions. Less than 30 proteins constitute BL21 IBs in baseline conditions, sharing close to 80% of them to the O157: H7 strain.

**Supplementary Note 7: Comparison to existing antimicrobial peptides (AMPs).** We analysed the sequences of our initial screen using the CAMP software prediction algorithm, which is trained to identify known AMPs^3^, and found that 90% were predicted to be antibacterial, irrespective of whether we found them to be active or not (65% were inactive). The Matthews Correlations Coefficient (MCC) with the measured activity was 0.1 at 12 μg mL^-1^ and 0.24 at 25 μg mL^-1^. Due to the conservation of hydrophobicity and charge of the mutant peptides, the CAMP prediction software also classifies our proline control peptides as antimicrobial. Together this indicates that the key properties of known AMPs, such as hydrophobicity and charge, captured by a machine learning algorithm are not sufficient to predict the antibacterial activity of aggregating peptides and that our peptides work by a mechanism not incorporated in the current prediction algorithms.

**Supplementary Note 8: Tolerance of mice to P2.** Given these positive results, we established the tolerance of Swiss mice to the peptide by performing a dose escalation experiment. We observed no acute adverse effects across the entire concentration range to the basic physiological and behavioural parameters of the animals upon intraperitoneal administration of up to 30 mg kg^-1^ P2. The parameters observed included body weight, food and water consumption, home cage activity and locomotion. We subsequently treated a cohort of 6 mice with daily injections at the maximum tolerated dose for 18 days without any apparent adverse effects. The mice were allowed to recover for 3 days, at which point they were sacrificed and a survey of the major organs was undertaken, which revealed no major morphological signs of toxicity (supplementary tables 6, 7 and supplementary figures 20, 21). In addition, an ELISA assay on the serum of these animals revealed no specific antibody response towards the P2 peptide (Figure 4H). A limited biodistribution study in healthy animals following injection (IP or urinary tract) of a single dose of 10 mg kg^-1^ FITC-labelled P2 revealed a clear distribution of fluorescent material for at least 3h after injection and a total clearance of the peptide after 24h (supplementary Figure 22).

**Supplementary Methods**

All strains used to generate the data in Supplementary Table 4 were from the Bacterial Culture Collection of Aptuit S.r.l. (Verona, Italy). MIC and MBC values were determined at Aptuit according to guideline M07-A10 of the Clinical and Laboratory Standards Institute (CLSI) and guideline M26-A1 of the National Committee for Clinical Laboratory Standards (NCCLS), respectively.

**Supplementary References**

1. Dana A, Tuller T. Mean of the typical decoding rates: a new translation efficiency index based on the analysis of ribosome profiling data. *G3 (Bethesda)* **5**, 73-80 (2014).

2. Shannon P*, et al.* Cytoscape: a software environment for integrated models of biomolecular interaction networks. *Genome Res* **13**, 2498-2504 (2003).

3. Waghu FH, Gopi L, Barai RS, Ramteke P, Nizami B, Idicula-Thomas S. CAMP: Collection of sequences and structures of antimicrobial peptides. *Nucleic Acids Res* **42**, D1154-1158 (2014).

4. Young LM*, et al.* Screening and classifying small-molecule inhibitors of amyloid formation using ion mobility spectrometry-mass spectrometry. *Nat Chem* **7**, 73-81 (2015).

5. Cremers CM*, et al.* Polyphosphate: A Conserved Modifier of Amyloidogenic Processes. *Mol Cell* **63**, 768-780 (2016).

6. Xu J*, et al.* Gain of function of mutant p53 by coaggregation with multiple tumor suppressors. *Nat Chem Biol* **7**, 285-295 (2011).

7. Wang G, Fersht AR. Multisite aggregation of p53 and implications for drug rescue. *Proc Natl Acad Sci U S A* **114**, E2634-E2643 (2017).

8. Ghosh S*, et al.* p53 amyloid formation leading to its loss of function: implications in cancer pathogenesis. *Cell death and differentiation*, (2017).

9. Soragni A*, et al.* A Designed Inhibitor of p53 Aggregation Rescues p53 Tumor Suppression in Ovarian Carcinomas. *Cancer cell* **29**, 90-103 (2016).

10. Wang M*, et al.* PaxDb, a database of protein abundance averages across all three domains of life. *Mol Cell Proteomics* **11**, 492-500 (2012).

11. Niwa T*, et al.* Bimodal protein solubility distribution revealed by an aggregation analysis of the entire ensemble of Escherichia coli proteins. *Proceedings of the National Academy of Sciences of the United States of America* **106**, 4201-4206 (2009).
