# Supplementary material for: Aggregating sequences that occur in many proteins constitute weak spots of bacterial proteostasis
Source: Nat Commun. 2018 Feb 28;9:866. doi: 10.1038/s41467-018-03131-0 (PMC5830399; doi:10.1038/s41467-018-03131-0)
Supplement: Supplementary file 2 — Description of Additional Supplementary Files [file 41467_2018_3131_MOESM2_ESM.docx]

**Description of Additional Supplementary Files**

File Name: Supplementary Data 1

Description: Proteins identified by deep MS in the IB fraction of E. coli O157: H7 after treatment with P2.

File Name: Supplementary Data 2

Description: Comparison of proteins identified by shortgun MS in the IB fraction of E. coli O157: H7 after toxic and non-toxic treatments

File Name: Supplementary Data 3

Description: Comparison of proteins identified by shortgun MS in the IB fraction of E. coli BL21 across conditions.
